# Effect of a Recycled Polyethylene Wax/Bio-Oil-Based Reactive Composite Rejuvenator on the Performance Balance Mechanism of Intermediate-Temperature Rejuvenation of Aged SBS-Modified Asphalt Binder

**DOI:** 10.3390/ma19163524

**Published:** 2026-08-19

**Authors:** Yijie Zhu, Junru Wang, Hongxiao Yang, Xiao Zhang

**Affiliations:** College of Civil Engineering, Taiyuan University of Technology, Taiyuan 030024, China; 2023002517@link.tyut.edu.cn (Y.Z.); 2023002511@link.tyut.edu.cn (J.W.); 2024002608@link.tyut.edu.cn (H.Y.)

**Keywords:** SBS-modified asphalt binder, composite rejuvenation, recycled polyethylene wax, infrared spectroscopy, microscopic mechanism

## Abstract

This study developed a composite rejuvenator comprising recycled polyethylene wax (PREW), waste cooking oil (WCO), and epoxidized soybean oil (ESO) activated by the tertiary amine catalyst BDMA to improve the intermediate-temperature rejuvenation of aged SBS-modified asphalt binder. The binder was subjected to combined rolling thin-film oven and pressure aging vessel aging. Conventional tests, rotational viscosity, bending beam rheometer, multiple stress creep recovery, fluorescence microscopy, and Fourier transform infrared spectroscopy were used to evaluate macroscopic, rheological, and microstructural properties. Aging hardened and embrittled the binder, increased softening point and viscosity, reduced penetration and ductility, and disrupted the polymer-rich phase. PREW reduced flow resistance and retained relatively high-temperature structural stability, whereas WCO improved flexibility and flowability, although excessive softening impaired high-temperature stability. ESO/BDMA treatment was accompanied by changes in oxygen-containing functional group-related absorption regions and improved apparent connectivity of the SBS-rich phase. Among the tested temperatures, 120 °C provided the best overall balance among the evaluated properties, satisfying low-temperature stress-relaxation requirements while limiting high-temperature creep deformation. These results identify 120 °C as the preferred treatment temperature for the PREW/WCO/ESO-BDMA rejuvenation system.

## 1. Introduction

Styrene–butadiene–styrene (SBS)-modified asphalt binder is extensively applied in heavy-duty and high-grade pavements owing to its desirable resistance to rutting at elevated temperatures, cracking at low temperatures, and its favorable elastic recovery [1,2]. During long-term service, however, thermal oxidation, repeated traffic loads, and environmental exposure jointly accelerate the deterioration of this material. Such deterioration involves both oxidative association within the asphalt fractions and progressive degradation of the SBS polymer phase. It has been reported that aging reduces penetration and ductility while increasing softening point and viscosity. Meanwhile, the polymer-rich phase becomes fragmented, its continuity is weakened, and SBS degradation becomes more severe, ultimately resulting in an imbalance between high- and low-temperature properties and a reduction in long-term service durability [3,4,5,6,7]. Therefore, developing effective rejuvenation strategies for severely aged SBS-modified asphalt binder is essential for enhancing the performance of recycled pavements, lowering maintenance costs, and promoting the sustainable recycling of road materials.

For the rejuvenation of aged SBS-modified asphalt binder, existing studies have mainly developed three approaches: physical rejuvenation, reactive rejuvenation, and composite rejuvenation. Previous studies have also demonstrated that different rejuvenation strategies may produce substantially different recovery effects in terms of rheological behavior, microscopic structure, and fatigue performance [8,9]. Physical rejuvenation is generally associated with light-component supplementation, colloidal-state regulation, and improved binder flowability. Cong et al. compared several rejuvenation methods for aged SBS-modified asphalt binder and reported that conventional rejuvenators could improve compositional balance, while the polymer-rich phase exhibited a comparatively smaller degree of morphological recovery [10]. Reactive rejuvenation emphasizes the involvement of active functional groups in the structural adjustment of aged SBS chain segments. Cao et al. reported rheological and structural changes consistent with enhanced connectivity of aged SBS chain segments by combining active components with bio-based rejuvenators [11], and further demonstrated from the perspective of all-component rejuvenation that high-quality rejuvenation benefits from the coordinated regulation of light components and the polymer-rich phase [12]. Han et al. developed reactive chain-extension rejuvenators and reported improvements in the structural characteristics and overall performance of rejuvenated binders [13]. Shi et al. and Xu et al. further indicated, from the perspectives of active rejuvenator design, synchronous rejuvenation pathways, and reactive rejuvenation mechanisms, that high-quality rejuvenation involves coordinated recovery of the colloidal structure of the aged matrix and the morphology of the polymer-rich phase [14,15,16,17]. Recent evidence further indicates that SBS modifier diffusion and interfacial evolution are closely associated with the performance recovery of aged SBS-modified asphalt systems [18]. Composite rejuvenation systems therefore attempt to integrate softening components, reactive components, and warm-mix technology to balance construction workability, low-temperature flexibility, and high-temperature stability.

In green composite rejuvenation systems, waste cooking oil, vegetable oils, and their derivatives have received increasing attention because of their wide availability, low cost, and relatively low environmental burden. Waste cooking oil (WCO) and vegetable-oil-based components have been reported to supplement light fractions, promote molecular mobility and component diffusion, and improve low-temperature flexibility and construction workability [19,20,21,22,23,24]. However, excessive use of oil-based rejuvenators may reduce high-temperature stability and increase sensitivity to secondary aging. To improve the balance achieved through physical softening, Zhang et al. proposed a WCO-MDI reactive rejuvenation pathway, and reported coordinated improvements in matrix-related properties and SBS-phase characteristics [25]. Li et al. developed a reactive warm-mix rejuvenator and demonstrated the feasibility of simultaneously achieving warm-mix energy saving and performance regulation under intermediate-temperature conditions [26].

In this study, polyethylene wax was incorporated mainly to achieve a warm-mix temperature-reduction effect. Compared with oil-based rejuvenators, polyethylene wax and recycled polyethylene wax materials have more specific functions in warm-mix rejuvenation systems. On the one hand, polyethylene wax can reduce the viscosity of asphalt binder in the medium- to high-temperature range and improve mixing and construction workability. On the other hand, wax crystalline domains formed during cooling may contribute to high-temperature structural support and moderate the strong softening response associated with oil-based rejuvenators. Previous studies have shown that composite rejuvenators containing virgin polyethylene wax and recycled polyethylene wax can be used for the warm-mix rejuvenation of aged SBS-modified asphalt binder and can affect the flowability, interfacial state, and service performance of rejuvenated asphalt binders [26,27,28]. In addition, PREW may form polar oxygen-containing functional groups during recycling, processing, or thermal oxidation. These groups may interact with polar components in aged asphalt through hydrogen bonding, polar adsorption, or esterification-related interactions, thereby improving the compatibility between the aged matrix and rejuvenating components [27,28,29]. Accordingly, PREW is considered a multifunctional component that may contribute to binder flow regulation, high-temperature structural support, and interfacial compatibility. These effects may provide a favorable mass-transfer environment for the dispersion and temperature-dependent interaction of ESO/BDMA components within the aged asphalt system.From an engineering perspective, binder-level performance recovery does not necessarily translate directly into equivalent improvement at the mixture scale, because aggregate–binder interactions, mixture volumetrics, and additional thermal history during production may influence the resulting mixture performance. Nevertheless, the use of waste-derived PREW and WCO provides a potential route for resource valorization and reduced reliance on virgin rejuvenating materials, while intermediate-temperature processing may reduce thermal energy demand. These potential engineering, environmental, and economic benefits require further verification through mixture-scale performance tests, life-cycle assessment, and cost analysis.

Based on the above studies, three issues merit further investigation in the intermediate-temperature rejuvenation of aged SBS-modified asphalt binder.

1. The functional contributions of individual components in composite rejuvenation systems require clearer experimental distinction. Severely aged SBS-modified asphalt binder simultaneously exhibits matrix hardening, light-component loss, reduced flowability, and degradation of the polymer-rich phase. A multi-indicator evaluation framework can therefore provide a more comprehensive assessment of rejuvenation performance than reliance on individual conventional indicators.
2. The intermediate-temperature operating window requires systematic identification. Lower treatment temperatures may favor physical softening and relatively slow temperature-dependent interactions, whereas higher temperatures may promote stronger component interactions together with increased thermal exposure. The coordinated evaluation of workability, low-temperature flexibility, and high-temperature stability can support the selection of an appropriate treatment range.
3. For composite systems containing PREW, WCO, and epoxy-type components, existing evidence has primarily emphasized individual materials and single-stage responses. The combined relationship among warm-mix viscosity regulation, oil-induced softening, and temperature-dependent physicochemical adjustment requires integrated evaluation through macroscopic properties, rheological behavior, FTIR band characteristics, and fluorescence morphology.

Accordingly, this study used SBS-modified asphalt binder subjected to combined RTFOT and PAV aging as the target binder and developed a composite rejuvenation system consisting of PREW, WCO, and ESO/BDMA. Progressive groups, namely P, PW, and PWEB, were designed to characterize the performance changes associated with the stepwise addition of different functional components. On this basis, rejuvenation temperatures of 110, 120, 130, and 140 °C were selected to systematically investigate the effects of the composite rejuvenator system on conventional properties, construction workability, low-temperature stress relaxation performance, high-temperature creep recovery behavior, and polymer-rich phase morphology. This study aims to characterize the performance contributions associated with PREW, WCO, and ESO/BDMA, identify an intermediate-temperature treatment window that balances warm-mix construction, low-temperature flexibility, and high-temperature deformation resistance, and provide binder-level evidence for the subsequent evaluation of recycled asphalt mixtures.

## 2. Materials and Methods

### 2.1. Raw Materials

In this study, laboratory-prepared SBS-modified asphalt binder was used as the base binder. Laboratory preparation was adopted to maintain consistent and traceable control over the base asphalt source, SBS content, oil fraction, stabilizer dosage, and thermal-shear history across all experimental groups. The base asphalt had a penetration of 68.4 (0.1 mm) at 25 °C, a softening point of 48.2 °C, a ductility of 112.0 cm at 15 °C, and a rotational viscosity of 435 mPa·s at 135 °C, as determined according to JTG E20-2011 [30].

The preparation procedure was as follows. First, a certain amount of base asphalt was heated to 180 °C until it was fully melted. Then, 4% SBS modifier and 4% oil fraction by mass were premixed and added to the base asphalt. The mixture was sheared using a high-speed shear mixer at 180 °C and 6500 r/min for 1 h to promote sufficient swelling and dispersion of SBS in the asphalt. Subsequently, 0.1% sulfur by mass of the base asphalt was added, and shearing was continued at 3000 r/min for 2 h to improve the stability of the modified system. The mixture was then stirred using a magnetic stirrer at 180 °C and 1600 r/min for 70 min to further enhance homogeneity. Finally, the prepared SBS-modified asphalt binder was conditioned in an oven at 150 °C for 1 h to allow structural stabilization before use. Its basic properties are listed in Table 1. The resulting binder satisfied the corresponding technical requirements and provided a controlled laboratory analogue of engineering SBS-modified asphalt binders. The test methods followed JTG E20-2011 [30], and the requirement values in Table 1 were adopted from JTG F40-2004 [31].

The PREW used in the rejuvenation system was recycled polyethylene wax, which was selected as a viscosity-regulating component under intermediate-temperature conditions and as a component contributing to the high-temperature structural stability of the composite system. WCO was obtained from waste cooking oil and, before use, was subjected to settling, filtration, and dehydration to remove impurities and free water, and it was introduced as a physical softening and diffusion-promoting component. ESO was epoxidized soybean oil, which served as the source of active epoxy groups in the composite rejuvenator. BDMA was used as a tertiary amine catalyst to facilitate the temperature-dependent reaction of ESO.

### 2.2. Preparation of Aged Asphalt Binder

To reproduce the aging process experienced by SBS-modified asphalt binder during mixing, construction, and in-service exposure, the binder was aged through a two-step laboratory procedure combining RTFOT and PAV. The RTFOT and PAV procedures followed test methods T 0610-2011 and T 0630-2011, respectively, as specified in JTG E20-2011 [30]. In the first step, the fresh binder was brought to a workable fluid condition and transferred into standard aging bottles. It was then treated at 163 °C for 85 min to represent the short-term thermal-oxidative effect occurring during mixing, transport, and paving. The residue obtained from RTFOT was further conditioned in a pressure aging vessel at 100 °C and 2.1 MPa for 20 h, which was intended to reproduce the long-term deterioration of asphalt binder under pavement service conditions. After this combined aging protocol, the binder showed a sharp decrease in penetration and ductility, together with clear increases in softening point and rotational viscosity, indicating severe hardening and embrittlement. Therefore, the binder aged successively by RTFOT and PAV was selected as the target material for subsequent rejuvenation tests. The detailed aging conditions are summarized in Table 2.

### 2.3. Rejuvenation Scheme and Experimental Groups

To identify the functional contributions of PREW, WCO, and ESO/BDMA in the composite rejuvenator system, eight groups of samples were prepared, including the fresh group, aged group, P group, PW group, and PWEB groups treated at different temperatures. The fresh group was used to characterize the performance of the original SBS-modified asphalt binder, while the aged group served as the control before rejuvenation. The P group contained PREW and was used to characterize the performance changes associated with PREW addition. The PW group was prepared by further adding WCO to the P system and was used to characterize the additional changes associated with WCO addition. The PWEB group was prepared by further introducing ESO and BDMA into the PREW/WCO system and was used to evaluate the additional performance response of the composite system. The progressive grouping design of P, PW, and PWEB was adopted to distinguish the functional contributions of different components in the composite rejuvenator. The P and PW groups were maintained at a fixed treatment temperature of 115 °C as component-comparison groups, whereas the PWEB groups were further evaluated at different temperature levels to examine the temperature-dependent performance response of the complete composite system.

The rejuvenation temperature levels were selected by jointly considering the temperature-dependent behavior of epoxy-containing reactive rejuvenation systems and the reduced processing-temperature range associated with warm-mix asphalt technology. Ma et al. [32] reported that the performance response of epoxy-type reactive rejuvenators for aged SBS-modified asphalt binder was closely related to treatment conditions. Cheraghian et al. [33] reviewed the design and performance of warm-mix asphalt technology and highlighted reduced mixing and construction temperatures while maintaining material performance. Li et al. [26] further demonstrated the feasibility of combining reactive rejuvenation with temperature reduction for aged SBS-modified asphalt binder under intermediate-temperature conditions. Accordingly, 110–140 °C was selected as the investigated intermediate-temperature range, and four temperature levels of 110, 120, 130, and 140 °C were arranged at 10 °C intervals. This design allowed the evolution of conventional properties, workability, rheological behavior, and microscopic characteristics to be compared across a continuous treatment-temperature range. The mix designs of all groups are listed in Table 3.

The dosages of all rejuvenating components were calculated based on the mass of aged SBS-modified asphalt binder. The dosage selection was guided by previous studies on polyethylene-wax-containing rejuvenation systems, WCO-based rejuvenation, and reactive rejuvenation of aged SBS-modified asphalt binder [22,25,26,27,29,32]. Within these literature-supported formulation concepts, preliminary screening focused on the material flow state and construction workability of the composite system. The preliminary screening was intended to identify a workable formulation rather than to perform a systematic dosage optimization.The resulting formulation consisted of 3.0% PREW and 8.0% WCO for the physical-regulation stage, together with 5.0% ESO and 0.5% BDMA for the subsequent composite treatment. These dosages were retained for all PWEB temperature groups so that the influence of treatment temperature could be evaluated under an identical material composition.

### 2.4. Preparation Procedure of Rejuvenated Asphalt Binder

**Figure 1:**
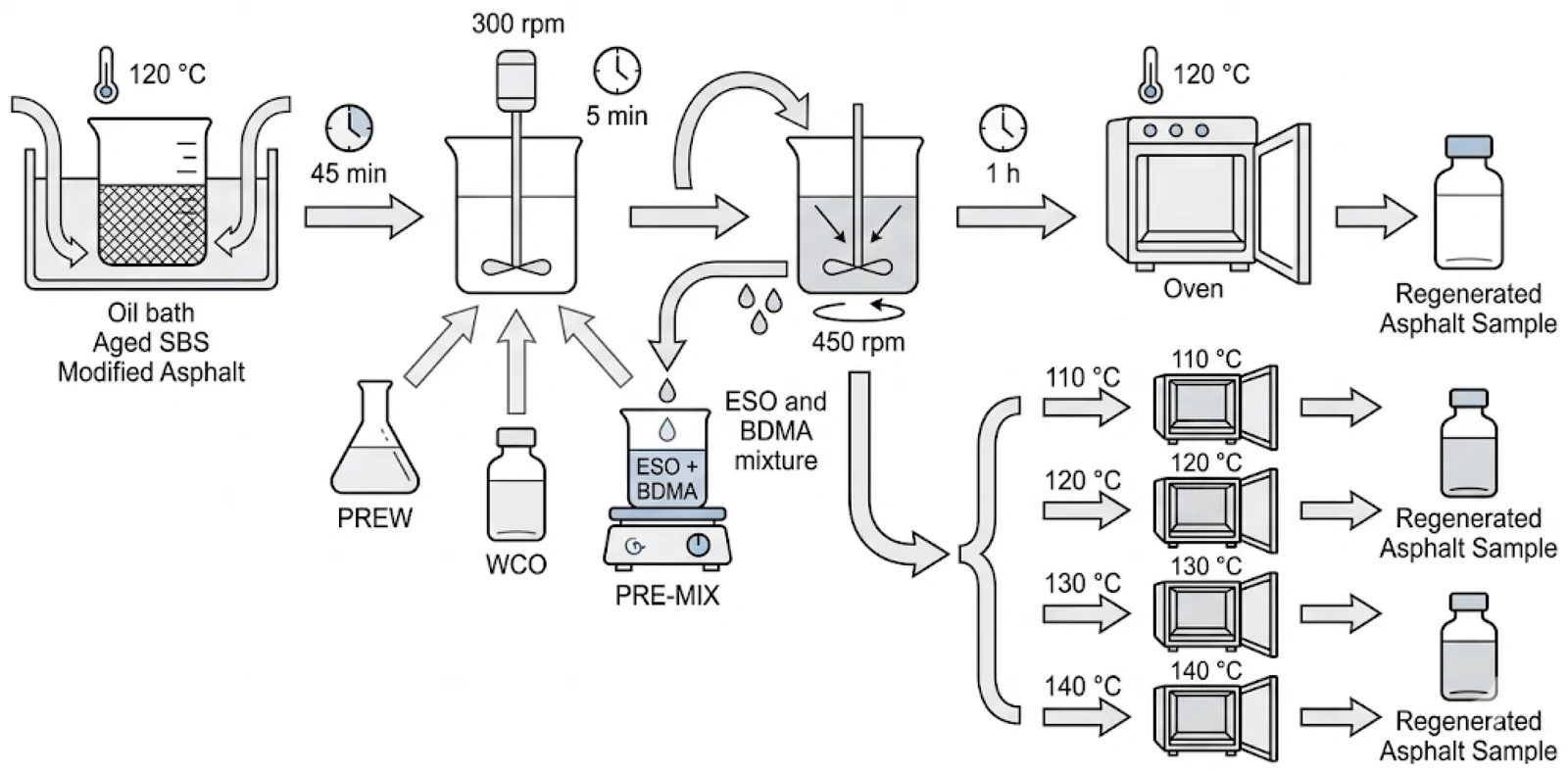
Preparation flowchart of the PREW/WCO/ESO-BDMA composite rejuvenator system.

The SBS-modified asphalt binder subjected to combined RTFOT and PAV aging was preheated in a constant-temperature oil bath at 120 °C and maintained for 45 min to ensure a uniform and stable flow state. Mechanical stirring was then initiated, and the sample was pre-stirred at 300 r/min for 5 min to eliminate local temperature differences and ensure a consistent initial state. As shown in Figure 1, the preparation sequence consisted of PREW incorporation for initial flow regulation, subsequent WCO addition for further softening and dispersion, and final introduction of ESO and BDMA as the reactive post-treatment components.

For the P group, only PREW pretreatment was performed. At the specified temperature, 3.0% PREW was slowly added in several portions. After addition, the stirring speed was increased to 450 r/min and maintained for 110 min. The sample was then conditioned in an oven at 120 °C for 1 h. For the PW group, 8.0% WCO was further added to the P group. The mixture was first stirred at 450 r/min for 40 min to ensure sufficient melting of PREW and uniform dispersion of WCO, followed by continued stirring for 70 min. The sample was then conditioned in the same manner for 1 h. The 110 min physical-treatment duration was kept consistent between the P and PW groups to provide comparable thermal and mixing histories. The selected duration was guided by the preparation procedures reported for wax- and oil-containing rejuvenation systems and by preliminary observations of material fluidity and mixing uniformity [26,27].

For the PWEB group, the ESO/BDMA post-treatment system was introduced after PREW and WCO pretreatment. Specifically, ESO and BDMA were premixed at room temperature for 3–5 min according to the designed proportion to form a homogeneous liquid mixture. The mixture was then slowly added dropwise into the main reactor, followed by continued stirring at the specified temperature and 450 r/min for 40 min to provide consistent contact and mixing between the ESO/BDMA components and the pretreated aged binder. Finally, the sample was conditioned in an oven at 120 °C for 1 h to provide a consistent post-treatment thermal history before subsequent testing. The 40 min treatment duration was selected with reference to reported epoxy-type reactive rejuvenation procedures and preliminary processing observations, while the subsequent 1 h conditioning step was applied uniformly to all rejuvenated groups to standardize the pre-test conditioning history [29,32].

For the PWEB temperature comparison, the ESO/BDMA treatment time was fixed at 40 min while the treatment temperature varied from 110 to 140 °C. Under this design, increasing temperature can alter molecular mobility, component interaction rates, and the extent of temperature-dependent reactions. Therefore, the performance differences among the PWEB temperature groups represent the combined response to treatment temperature and the associated temperature-dependent interaction extent. This coupled effect was considered when interpreting the temperature-dependent results.

### 2.5. Test Methods

Penetration at 25 °C was measured under a load of 100 g for 5 s using Method T 0604-2025 of JTG 3410-2025 [34]. The softening point was determined using the ring-and-ball method according to Method T 0606-2025 of the same standard, and ductility at 5 °C was measured according to Method T 0605-2025. Rotational viscosity was measured at 135 and 175 °C.

Rotational viscosity was measured using an NDJ-1C rotational viscometer (Shanghai Changji Geological Instrument Co., Ltd., Shanghai, China) in accordance with Method T 0625-2011 of the aforementioned standard. A No. 27 spindle was used at a rotational speed of 50 r/min. Before testing, the asphalt binder samples were conditioned at the corresponding test temperature for 90 min to achieve a stable testing temperature. Rotational viscosity was subsequently measured at 135 and 175 °C.

Low-temperature rheological tests were conducted using an SYD-0627 bending beam rheometer (Shanghai Changji Geological Instrument Co., Ltd., Shanghai, China) at −12 °C and −18 °C in accordance with Method T 0627-2011 of the aforementioned standard. Dynamic shear rheometer (DSR) temperature-sweep tests were performed using a Discovery HR-10 dynamic shear rheometer (TA Instruments, New Castle, DE, USA) in accordance with Method T 0628-2011 of the same standard. A 25 mm parallel-plate geometry with a gap of 1.0 mm was used. The test temperature ranged from 40 to 90 °C at 5 °C intervals, with an angular frequency of 10 rad/s.

Multiple stress creep recovery (MSCR) tests were conducted using the same dynamic shear rheometer at 64 °C in accordance with Method T 0647-2025 of the aforementioned standard. Stress levels of 0.1 and 3.2 kPa were applied, with 10 creep–recovery cycles conducted at each stress level. Each cycle consisted of 1 s of loading followed by 9 s of recovery.

Fluorescence microscopy was performed using a fluorescence microscope manufactured by Xi’an Cewei Optoelectronic Technology Co., Ltd. (Xi’an, China) at 100-fold magnification. The acquired fluorescence images were processed using Image-Pro Plus software.

To ensure the reliability of the test results, no fewer than three parallel tests were conducted for each group in the conventional property, rotational viscosity, BBR, and DSR-related tests, and the arithmetic mean was used as the final result. When the parallel test results showed large dispersion, new samples were prepared and retested to reduce the influence of random errors on the analysis. In this study, data analysis was conducted using a combination of recovery ratio calculation and comprehensive balance evaluation.

For indicators where a higher value indicates better performance, such as penetration, ductility, and *m*-value, the recovery ratio was calculated as follows:

(1) $$R_{i}=\frac{X_{i}^{\mathrm{reg}}-X_{i}^{\mathrm{aged}}}{X_{i}^{\mathrm{fresh}}-X_{i}^{\mathrm{aged}}}\times 100\%,$$

where $R_{i}$ is the recovery ratio of the *i*-th indicator, $X_{i}^{\mathrm{reg}}$ is the test value of the rejuvenated group, $X_{i}^{\mathrm{aged}}$ is the test value of the aged group, and $X_{i}^{\mathrm{fresh}}$ is the test value of the fresh group. A higher $R_{i}$ indicates a higher recovery degree of the corresponding indicator.

For indicators where values closer to those of the fresh asphalt binder are preferred, such as softening point, rotational viscosity, and *S*-value, a deviation recovery ratio was used to avoid bias caused by judging performance only from the increasing or decreasing direction:

(2) $$R_{i}^{\prime }=\left(1-\frac{\left|X_{i}^{\mathrm{reg}}-X_{i}^{\mathrm{fresh}}\right|}{\left|X_{i}^{\mathrm{aged}}-X_{i}^{\mathrm{fresh}}\right|}\right)\times 100\%.$$

A higher $R_{i}^{\prime }$ indicates that the rejuvenated group is closer to the level of the fresh asphalt binder.

On this basis, to comprehensively evaluate the balance between high- and low-temperature properties of materials under different rejuvenation temperatures and mix designs, a dimensionless normalization method was used to construct a comprehensive balance index. After standardizing the recovery ratios of each indicator, the comprehensive evaluation value was calculated as follows:

(3) $$B=\sum_{i=1}^{n}w_{i}Z_{i},$$

where *B* is the comprehensive balance index, $Z_{i}$ is the standardized value of the *i*-th indicator, and $w_{i}$ is the corresponding weight, satisfying $\sum_{i=1}^{n}w_{i}=1$. In this study, all indicators were assigned equal weights because no subjective weighting was introduced. A higher *B* value indicates a better comprehensive balance effect of the rejuvenation scheme in terms of conventional properties, construction workability, and high- and low-temperature rheological recovery.

## 3. Results and Discussion

### 3.1. Evolution of Conventional Physical Properties and Construction Flowability

#### 3.1.1. Variation in the Three Conventional Indicators

Three independent parallel determinations were conducted for each quantitative test, and the results are expressed as the arithmetic mean ± standard deviation (SD). Overall differences among the experimental groups were evaluated using one-way analysis of variance (ANOVA), with the significance level set at p<0.05.

Table 4 presents the results of the three conventional property tests for the fresh group, aged group, P group, PW group, and PWEB groups treated at different temperatures. The mean values and corresponding standard deviations of the parallel measurements are summarized in the table.

**Figure 2:**
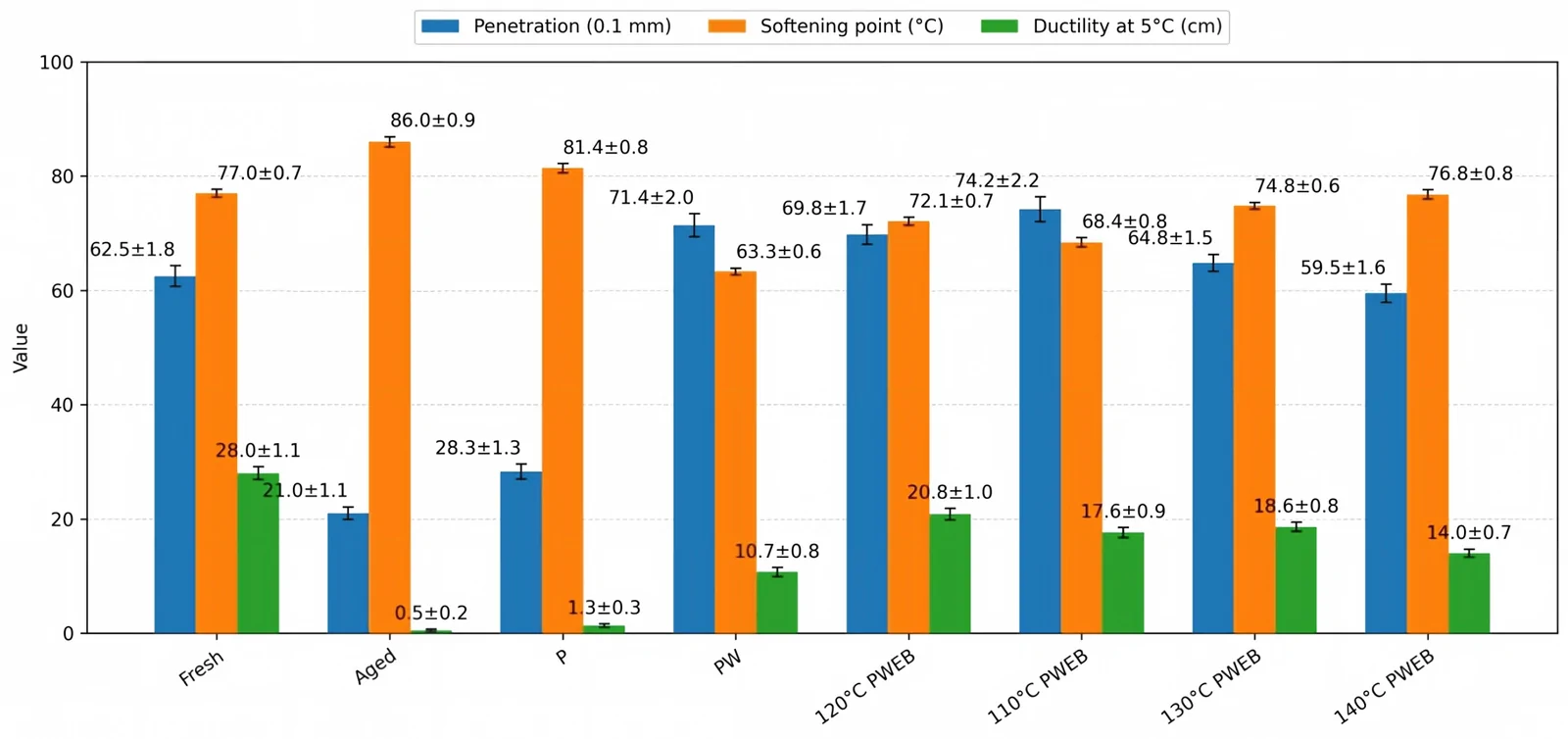
Conventional properties of different asphalt binder groups. Error bars represent standard deviations of three parallel determinations.

As shown in Figure 2, thermal oxidative aging produced pronounced changes in the three conventional properties of the SBS-modified asphalt binder. Compared with the fresh group, the penetration at 25 °C of the aged group decreased from 62.5 to 21.0 (0.1 mm), the softening point increased from 77.0 °C to 86.0 °C, and the ductility at 5 °C sharply decreased from 28.0 cm to 0.5 cm. One-way ANOVA indicated significant overall differences among the experimental groups for penetration, softening point, and ductility (p<0.001 for all three indicators). These results indicate that the binder underwent pronounced hardening and embrittlement after combined aging, accompanied by a severe loss of low-temperature deformation capacity. From a physicochemical perspective, this response is consistent with enhanced association of the relatively polar and high-molecular-weight fractions in the aged asphalt matrix, reduced molecular mobility, and deterioration of the polymer-rich phase. These combined changes increase the rigidity of the colloidal structure and restrict deformation at low temperature.

In terms of the composite rejuvenation results, after the addition of PREW, the penetration and ductility of the P group recovered to 28.3 and 1.3 cm, respectively, while the softening point decreased to 81.4 °C. The simultaneous increase in penetration and decrease in softening point indicate an increase in the mobility of the aged binder after PREW incorporation. The relatively high softening point of the P group also reflects the retention of a comparatively stiff high-temperature physical state. This response may be associated with the temperature-dependent melting and dispersion of PREW, which facilitates flow within the aged matrix while the wax-containing phase contributes to the physical organization of the binder during cooling. Judging from the recovery amplitudes of penetration and ductility, the overall improvement in the P group was limited. The low ductility value further indicates that the aged system retained a highly constrained molecular state and a relatively rigid colloidal structure after PREW treatment.

After WCO was further added on the basis of the P group, the penetration of the PW group increased to 71.4, and the ductility increased to 10.7 cm. The large increases in penetration and ductility indicate substantially enhanced molecular mobility and deformation capacity after WCO incorporation. The oil-rich fraction may increase the mobility of the maltene-like phase, enlarge the effective free volume of the aged binder, and weaken the physical association among relatively rigid asphalt fractions. Such changes provide a more mobile colloidal environment for deformation and component diffusion. The softening point of the PW group decreased to 63.3 °C, which was considerably lower than those of the fresh group and the P group. The combination of high penetration, increased ductility, and a low softening point therefore characterizes a strongly softened physical state. The enhanced molecular mobility improves flexibility and flowability, while the reduced stiffness of the continuous phase corresponds to lower high-temperature structural resistance.

After ESO and BDMA were introduced into the PREW/WCO system, the PWEB groups exhibited a temperature-dependent redistribution of the three conventional properties. The 120 °C PWEB group exhibited a penetration of 69.8, a softening point of 72.1 °C, and a ductility of 20.8 cm. Compared with the PW group, the increase in softening point together with the higher ductility indicates a shift from the strongly softened PW state toward a more balanced combination of flexibility and thermal resistance. This response may be associated with temperature-dependent interactions among ESO/BDMA, the oil-rich phase, and the aged asphalt matrix, which modify molecular mobility and the physical organization of the composite system.

A further comparison among the PWEB groups treated at different temperatures showed that the 110 °C group had the highest penetration of 74.2 and a softening point of 68.4 °C. These values characterize a comparatively mobile and softened state at the lower treatment temperature. The softening point of the 130 °C group increased to 74.8 °C, accompanied by a reduction in penetration and a moderate decrease in ductility relative to the 120 °C group. The softening point of the 140 °C group further increased to 76.8 °C, whereas its penetration decreased to 59.5 and its ductility decreased to 14.0 cm. This evolution reflects progressively restricted molecular mobility and a more rigid physical state as the treatment temperature increased. The higher thermal exposure may enhance temperature-dependent component interactions and promote a more compact organization of the binder system.

Overall, as the rejuvenation temperature increased from 110 °C to 140 °C, the penetration gradually decreased, the ductility first increased and then decreased, and the softening point continuously increased. Among the four PWEB temperature groups, the 120 °C treatment provided the closest simultaneous coordination of penetration, softening point, and ductility toward the fresh-binder level. This conclusion is based on the combined response of the three conventional indicators, while the subsequent rheological tests provide further evaluation of the high- and low-temperature performance balance.

#### 3.1.2. Rotational Viscosity Characteristics

The rotational viscosity results were obtained from three independent parallel determinations and are expressed as mean ± SD. Overall differences among the experimental groups at each test temperature were evaluated using one-way ANOVA with a significance level of p<0.05.

**Figure 3:**
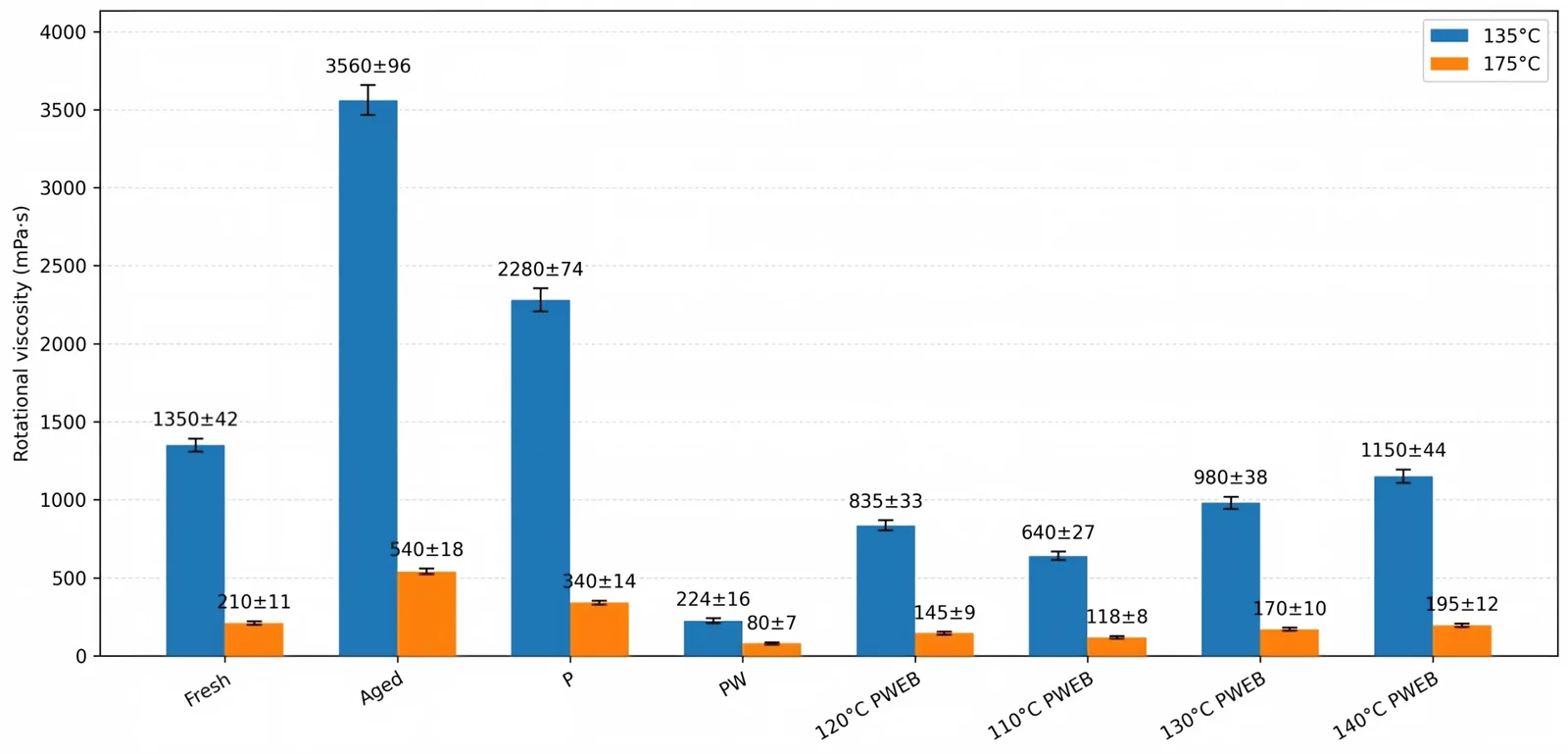
Rotational viscosity of different asphalt binder groups. Error bars represent standard deviations of three parallel determinations.

As shown in Figure 3, thermal oxidative aging markedly increased the flow resistance of the SBS-modified asphalt binder within the construction temperature range. Compared with the fresh group, the rotational viscosity of the aged group at 135 °C increased from 1350 ± 42 mPa·s to 3560 ± 96 mPa·s, and that at 175 °C increased from 210 ± 11 mPa·s to 540 ± 18 mPa·s. One-way ANOVA indicated significant overall differences among the experimental groups at both 135 °C and 175 °C (p<0.001). The viscosity increase is consistent with reduced molecular mobility and stronger physical association within the aged binder, resulting in greater resistance to shear flow during processing.

After PREW was added, the rotational viscosities of the P group at 135 °C and 175 °C decreased to 2280 ± 74 mPa·s and 340 ± 14 mPa·s, respectively. The decrease in viscosity indicates increased flowability relative to the aged group and is consistent with enhanced molecular mobility after PREW incorporation. The magnitude of the viscosity reduction remained moderate, which agrees with the relatively high softening point observed for the P group. Together, these results characterize a state with improved processability and retained high-temperature stiffness.

After WCO was further introduced into the PW group, the rotational viscosity decreased sharply. The viscosities at 135 °C and 175 °C were 224 ± 16 mPa·s and 80 ± 7 mPa·s, respectively. This pronounced viscosity reduction is consistent with a substantial increase in molecular mobility after incorporation of the oil-rich component. The increased fluidity may arise from a more mobile continuous phase and reduced physical association among the relatively rigid fractions of the aged binder. The viscosity response agrees with the simultaneous increase in penetration and decrease in softening point observed for the PW group.

Compared with the PW group, the PWEB group exhibited an increase in rotational viscosity after the introduction of ESO and BDMA. For the 120 °C PWEB group, the rotational viscosities at 135 °C and 175 °C were 835 ± 33 mPa·s and 145 ± 9 mPa·s, respectively. The increase relative to the PW group indicates a reduction in the highly mobile state produced by WCO, while the viscosity remained below the fresh-binder level at both test temperatures. This intermediate flow state is consistent with the simultaneous recovery of softening point and ductility observed in the conventional-property tests. The combined response suggests a redistribution of molecular mobility within the composite system after ESO/BDMA treatment.

The rotational viscosity results of the PWEB groups treated at different rejuvenation temperatures further indicate that temperature had a pronounced influence on the flow behavior of the system. The 110 °C group showed rotational viscosities of 640 ± 27 mPa·s and 118 ± 8 mPa·s at 135 °C and 175 °C, respectively. These relatively low viscosities characterize a highly mobile binder state at the lower treatment temperature.

As the rejuvenation temperature increased to 130 °C and 140 °C, the rotational viscosities increased to 980 ± 38 and 1150 ± 44 mPa·s at 135 °C and to 170 ± 10 and 195 ± 12 mPa·s at 175 °C, respectively, gradually approaching the level of the fresh group. The progressive increase in viscosity with treatment temperature indicates a gradual restriction of molecular mobility. Within the constant 40 min treatment period adopted for the PWEB groups, higher temperatures also correspond to greater molecular mobility during treatment and stronger temperature-dependent component interactions. Consequently, the observed viscosity evolution represents the combined influence of treatment temperature and the associated extent of physicochemical interaction within the composite system.

Overall, the rotational-viscosity results show a progression from the highly viscous aged state, through PREW-induced flow regulation and WCO-associated strong softening, to an intermediate viscosity range after ESO/BDMA treatment. Among the investigated PWEB groups, the 120 °C treatment retained substantial construction flowability while moving away from the strongly softened PW state. Together with the three conventional properties, these results support further evaluation of 120 °C as a candidate temperature for achieving a balanced binder response, which is examined using the subsequent BBR, DSR, and MSCR results.

### 3.2. High- and Low-Temperature Rheological Behavior

#### 3.2.1. Analysis of Low-Temperature Stress Relaxation Performance Based on BBR Test

Among the low-temperature rheological indicators, the creep stiffness *S* is used to characterize the deformation resistance stiffness of the material under low-temperature loading. A lower *S* value indicates lower low-temperature stiffness and a relatively lower risk of brittle cracking. The creep rate *m* is used to characterize the ability of the material to release thermal shrinkage stress. A higher *m* value indicates stronger low-temperature stress relaxation capacity. In general, a lower *S* value together with a higher *m* value corresponds to better low-temperature cracking resistance.

**Figure 4:**
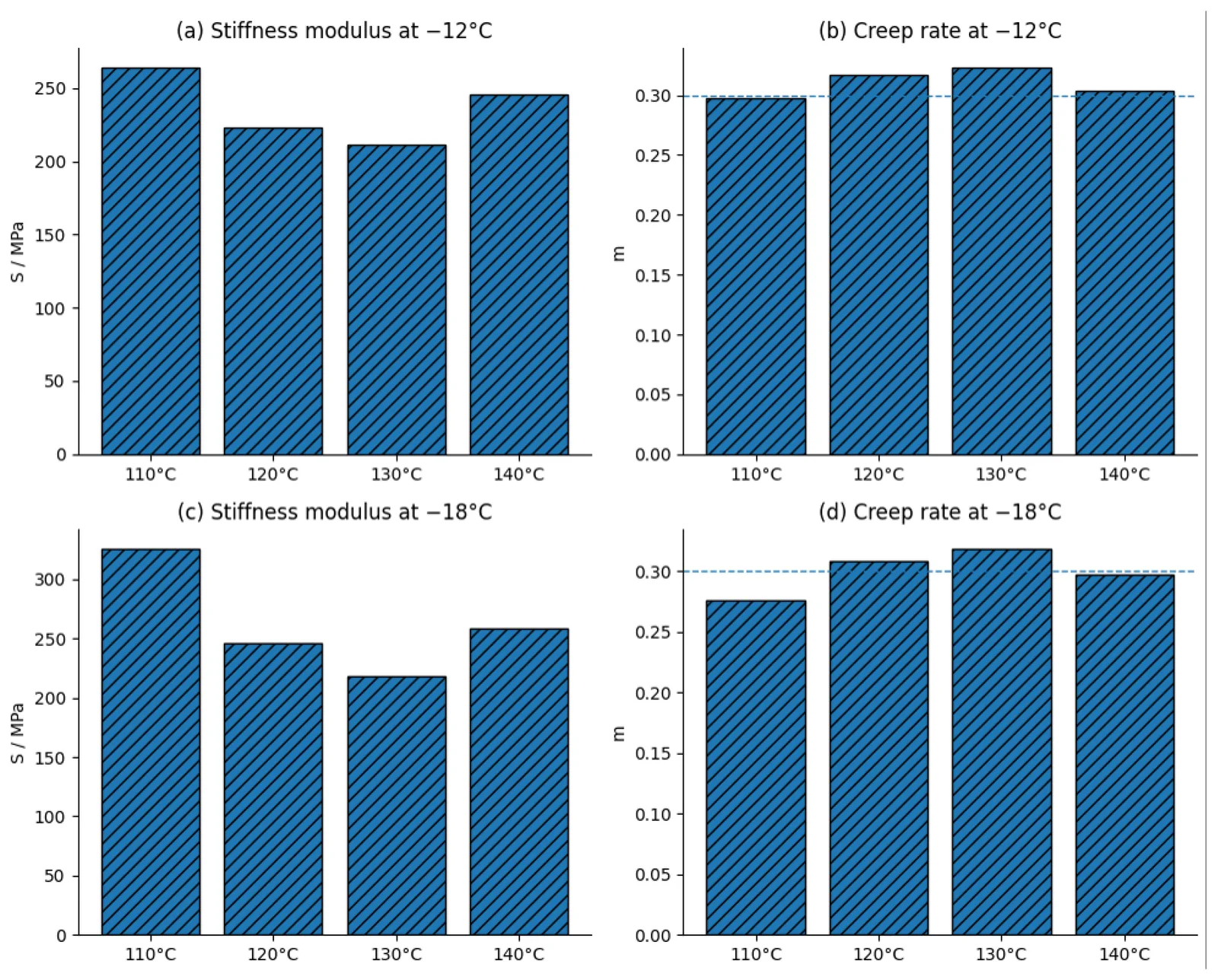
Comparison of BBR low-temperature rheological indicators of PWEB groups treated at different temperatures.

As shown in Figure 4, the low-temperature performance of the PWEB groups treated at different rejuvenation temperatures exhibited evident temperature sensitivity.

At 110 °C, the *S* values of the specimens at −12 °C and −18 °C were 264 MPa and 326 MPa, respectively, while the corresponding *m* values were 0.298 and 0.276. The relatively high stiffness and low *m* values characterize a comparatively restricted low-temperature molecular state, indicating limited stress relaxation under this treatment condition. When the rejuvenation temperature increased to 120 °C, the *S* values at −12 °C and −18 °C decreased to 223 MPa and 246 MPa, respectively, while the *m* values increased to 0.317 and 0.309. This simultaneous decrease in *S* and increase in *m* reflects enhanced low-temperature deformability and stress relaxation. Such a response may be associated with increased molecular mobility and more effective redistribution of the rejuvenating components within the aged binder.

After the temperature was further increased to 130 °C, the PWEB group showed better performance in terms of the individual BBR indicators. The *S* values at −12 °C and −18 °C were 211 MPa and 218 MPa, respectively, both of which were relatively low among the four temperature groups. The corresponding *m* values were 0.323 and 0.319, indicating the highest low-temperature flexibility and stress-relaxation response among the investigated temperature groups. The further increase in treatment temperature may promote component diffusion and molecular rearrangement, thereby reducing the effective stiffness of the low-temperature binder structure. When the temperature increased to 140 °C, the *S* values at −12 °C and −18 °C increased again to 246 MPa and 258 MPa, respectively, while the *m* values decreased to 0.304 and 0.297. In particular, the *m* value at −18 °C was 0.297, corresponding to a reduction in low-temperature stress-relaxation capacity relative to the 120 °C and 130 °C groups. The higher thermal exposure may promote a more constrained structural state after cooling and alter the balance between the oil-rich phase and the more rigid binder fractions.

Overall, the BBR indicators of the PWEB system first improved and then declined with increasing rejuvenation temperature, indicating the presence of an appropriate temperature window for low-temperature performance. Among the four groups, the 130 °C treatment provided the most favorable individual BBR response. The temperature-dependent evolution suggests that the low-temperature rheological state reflects the combined effects of component mobility, diffusion, and structural organization during treatment. The subsequent Multiple Stress Creep Recovery (MSCR) Test results showed that the peak strain and residual strain of the 130 °C group were substantially higher than those of the 120 °C group. Therefore, the 130 °C group is favorable from the perspective of the individual BBR indicators, whereas the 120 °C group shows a more coordinated response when conventional properties, rotational viscosity, low-temperature rheology, and high-temperature creep behavior are considered together.

#### 3.2.2. Analysis of High-Temperature Rheological Properties Based on DSR Temperature Sweep

To further evaluate the high-temperature viscoelastic response characteristics of asphalt binders at different rejuvenation stages and different rejuvenation temperatures, DSR temperature sweep tests were conducted to measure the complex shear modulus $G^{*}$, phase angle δ, and rutting factor $G^{*}$/sinδ of each group. In general, $G^{*}$ reflects the overall resistance of the material to shear deformation, δ represents the relative proportion of viscous and elastic components, and $G^{*}$/sinδ is used to characterize the high-temperature resistance to permanent deformation. Higher $G^{*}$ and $G^{*}$/sinδ values generally indicate stronger high-temperature rutting resistance, while a lower δ value indicates a more pronounced elastic response.

**(1) High-temperature rheological response during the synergistic rejuvenation stage**

**Figure 5:**
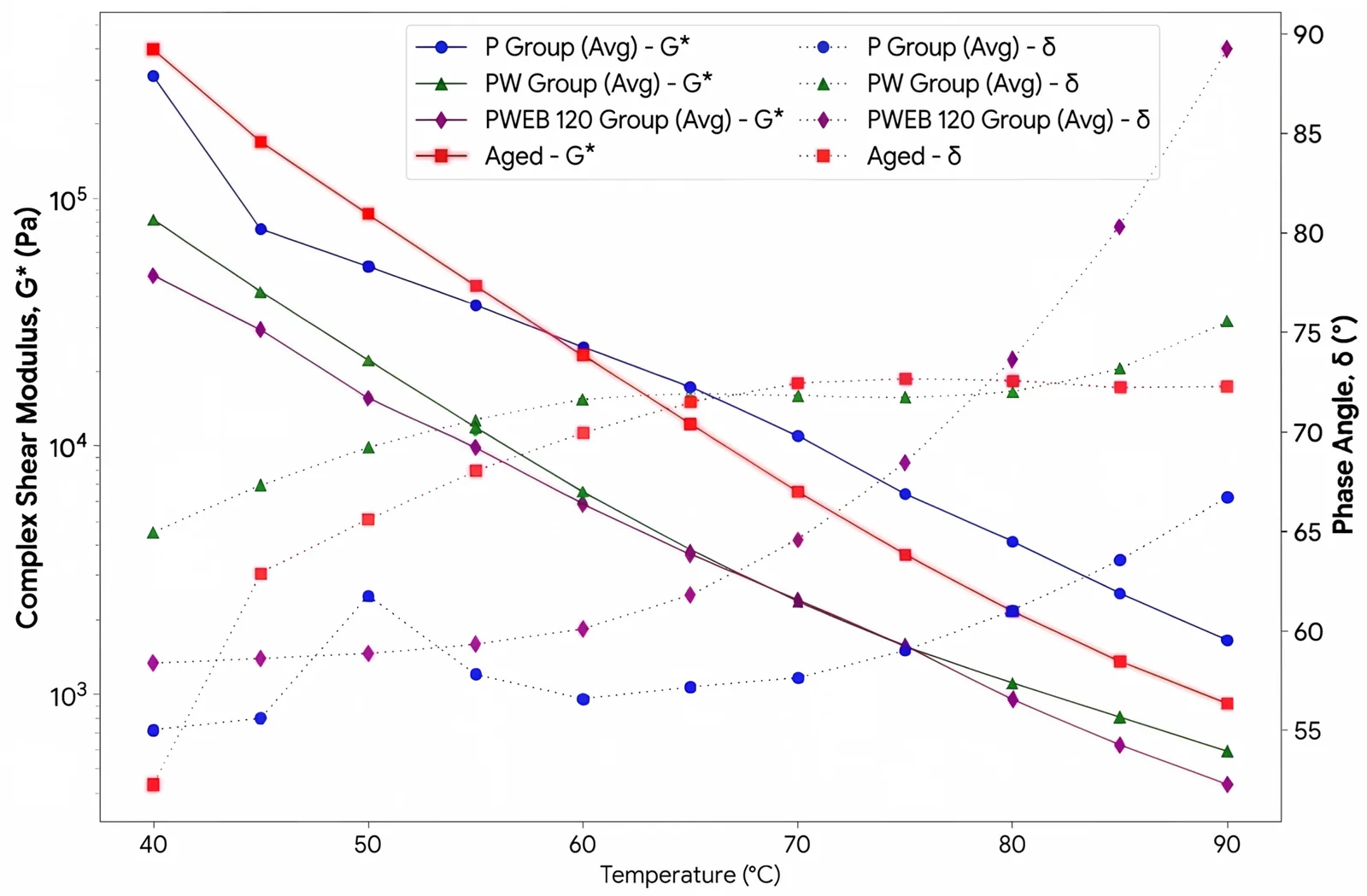
Variations in complex shear modulus $G^{*}$ and phase angle δ of the synergistic rejuvenation system.

**Figure 6:**
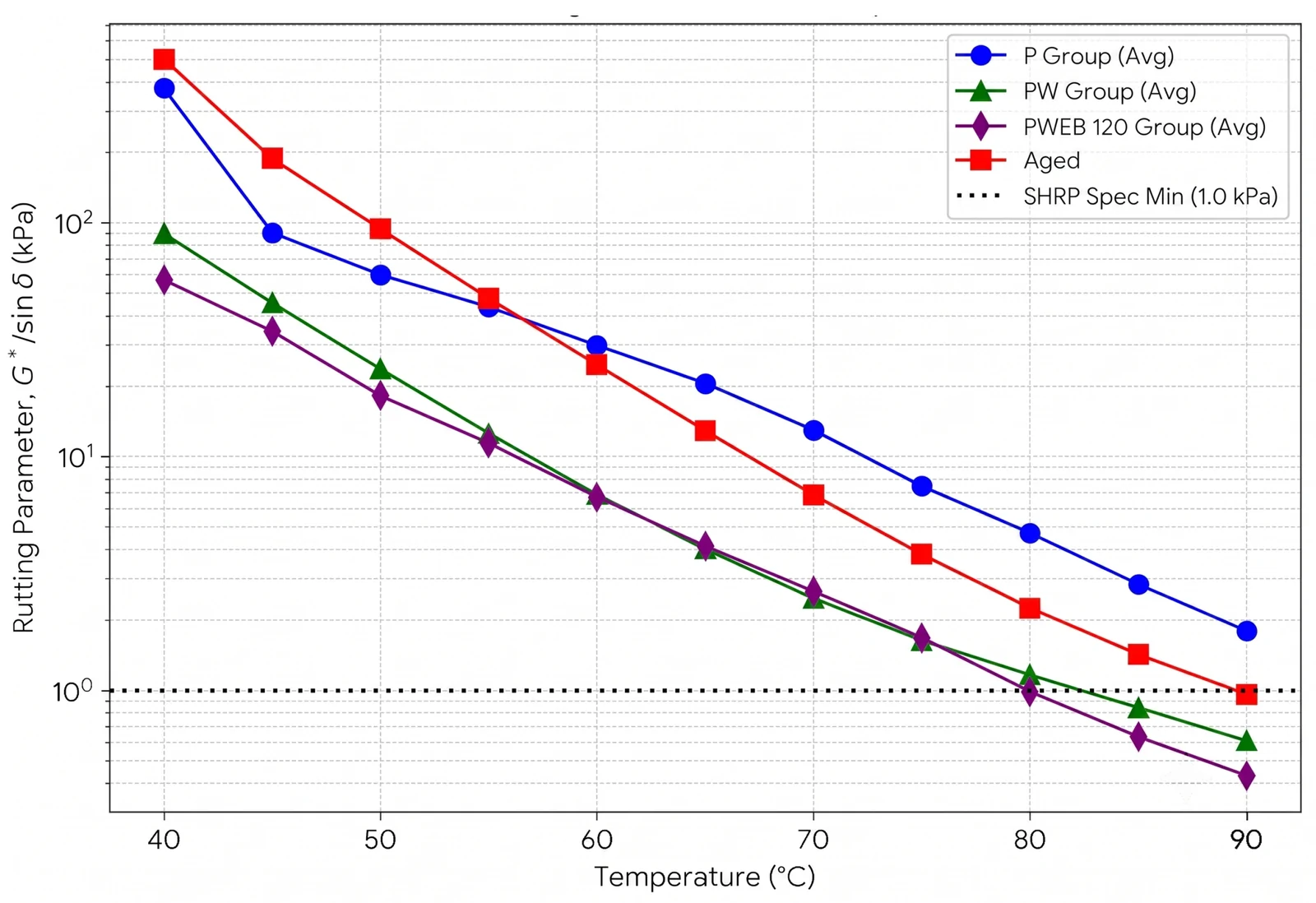
Variation in rutting factor $G^{*}$/sinδ of the synergistic rejuvenation system.

As shown in Figure 5 and Figure 6, the asphalt binders at different rejuvenation stages showed clear differences in high-temperature rheological behavior. The aged group generally exhibited relatively high $G^{*}$ and $G^{*}$/sinδ values, which was associated with material hardening caused by thermal oxidative aging. This high-modulus state primarily reflects the increased rigidity and restricted molecular mobility of the aged binder and should therefore be interpreted together with its severe loss of flexibility. After PREW was added, the $G^{*}$ and $G^{*}$/sinδ values of the P group were adjusted compared with those of the aged group, showing reduced aging-induced rigidity while retaining a comparatively high level of high-temperature shear resistance. This rheological response is consistent with the combined viscosity-reducing and wax-related physical organization observed from the conventional-property and rotational-viscosity results.

After WCO was further introduced into the PW group, $G^{*}$ and $G^{*}$/sinδ decreased markedly, indicating a more viscous and deformable high-temperature rheological state. The oil-rich component may increase the mobility of the continuous phase and reduce the physical association among rigid fractions, which is consistent with the lower softening point and rotational viscosity of the PW group.

Compared with the PW group, the PWEB group exhibited a recovery in high-temperature rheological indicators after the introduction of ESO and BDMA. The increase in $G^{*}$ and $G^{*}$/sinδ relative to the PW group indicates that the highly softened rheological state was shifted toward greater resistance to shear deformation. This change may reflect temperature-dependent physicochemical interactions among ESO/BDMA, WCO, PREW, and the aged binder, together with a redistribution of molecular mobility within the composite system. The DSR results therefore provide rheological evidence of a change in the structural state of the binder rather than direct evidence of a specific chemical reconstruction pathway.

**(2) High-temperature rheological evolution of PWEB groups at different rejuvenation temperature levels**

**Figure 7:**
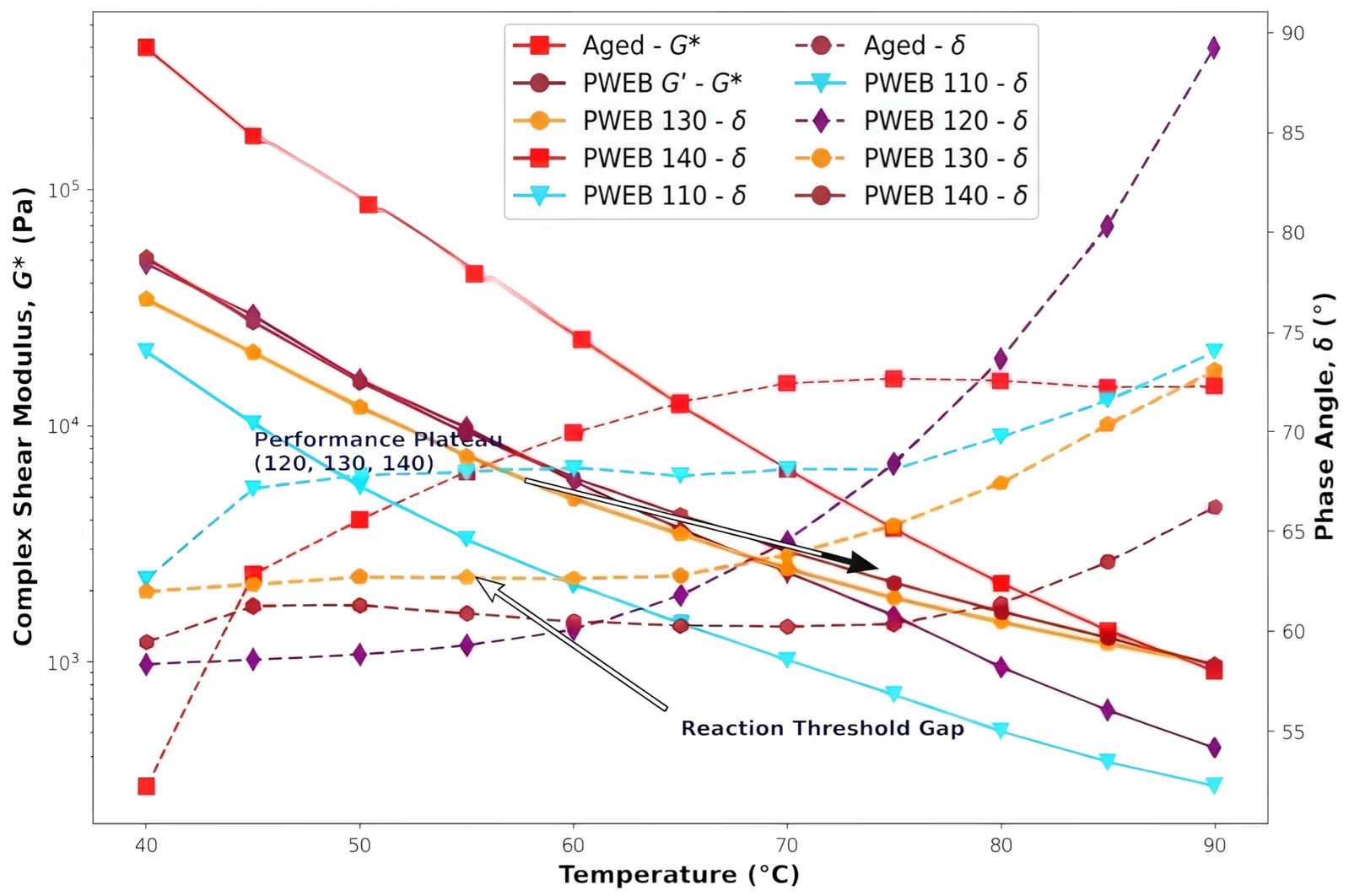
Variations in complex shear modulus $G^{*}$ and phase angle δ of PWEB groups treated at different temperatures.

**Figure 8:**
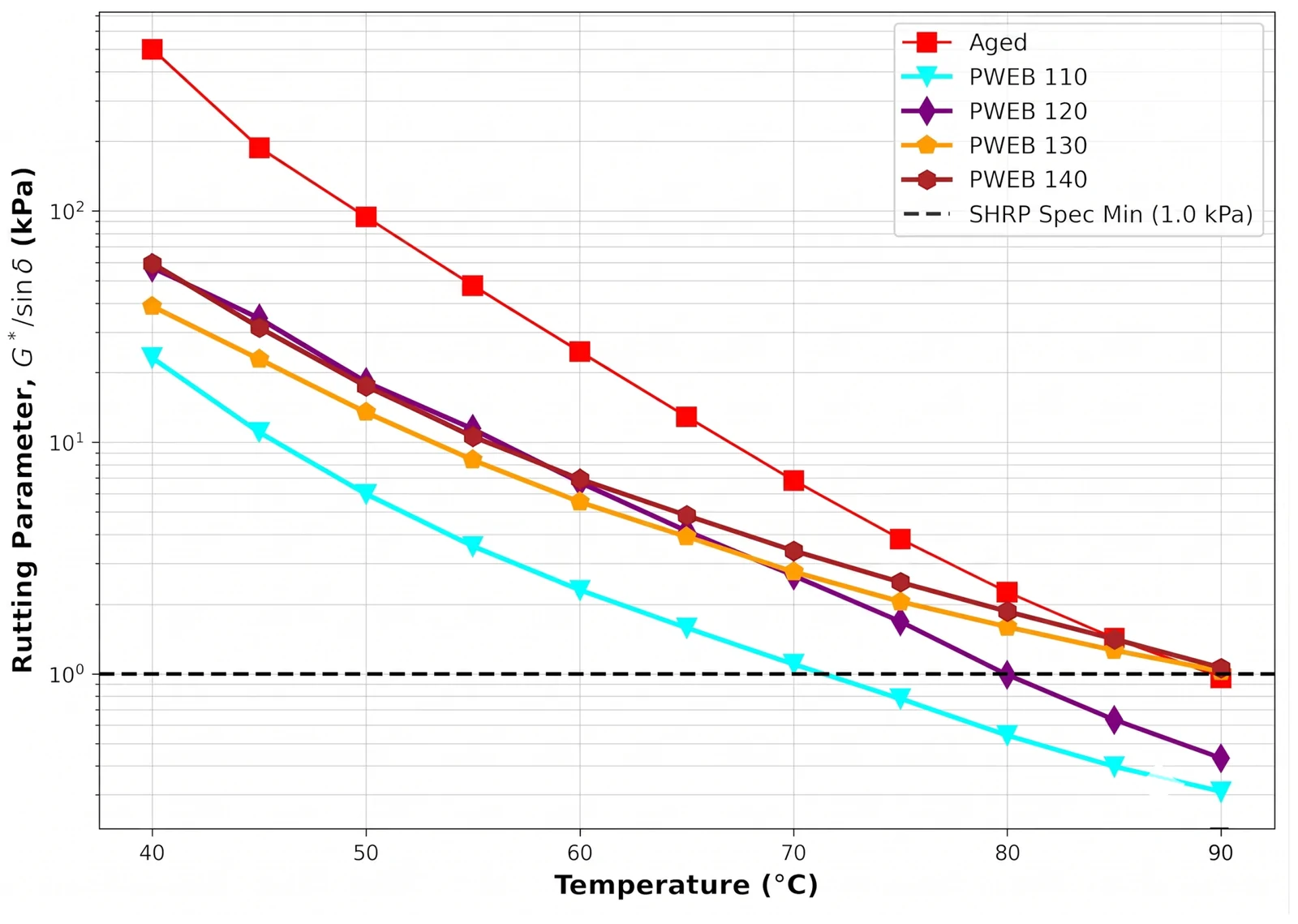
Variation in rutting factor $G^{*}$/sinδ of PWEB groups treated at different temperatures.

As shown in Figure 7 and Figure 8, the complex shear modulus $G^{*}$ and rutting factor $G^{*}$/sinδ of all temperature groups continuously decreased with increasing test temperature, indicating that the high-temperature shear deformation resistance of each group gradually weakened. This common trend reflects the increase in molecular mobility and viscous response of asphalt binder as the DSR test temperature rises.

At the same test temperature, when the rejuvenation temperature increased from 110 °C to 120 °C, both $G^{*}$ and $G^{*}$/sinδ increased noticeably, especially within the test temperature range of 40–65 °C, where the difference between the two groups was relatively pronounced. This change indicates greater high-temperature shear resistance of the 120 °C group relative to the 110 °C group. Meanwhile, the phase angle of the 120 °C group was generally lower than that of the 110 °C group within this test temperature range, indicating a relatively enhanced elastic response. The combined changes in modulus and phase angle may be associated with stronger temperature-dependent interactions and a more constrained rheological structure formed during the 120 °C treatment.

When the rejuvenation temperature further increased to 130 °C and 140 °C, the differences in $G^{*}$ and $G^{*}$/sinδ among the groups within the range of 40–65 °C became relatively smaller, showing a reduced rate of change in the high-temperature rheological response above 120 °C. As the test temperature increased above 70 °C, the differences among the temperature groups increased again. Among them, the 130 °C and 140 °C groups showed relatively higher rutting factors, while the rutting factor of the 120 °C group decreased more rapidly. The higher values observed for the 130 °C and 140 °C groups indicate greater resistance to high-temperature shear deformation in this test range, while their overall performance requires joint consideration of the corresponding low-temperature response.

Overall, increasing the rejuvenation temperature from 110 °C to 120 °C produced a clear increase in the high-temperature rheological indicators, while the subsequent changes from 120 °C to 140 °C became more temperature-range dependent. The 130 °C and 140 °C treatments exhibited advantages in rutting-related indicators at the higher DSR test temperatures, whereas the 120 °C group provided a more moderate rheological state for subsequent comprehensive performance evaluation.

#### 3.2.3. Analysis of High-Temperature Creep Recovery Characteristics Based on MSCR Test

To quantitatively characterize the recovery capacity, the recovery rate was calculated using the peak strain $\varepsilon_{\max}$ at 1.0 s and the residual strain $\varepsilon_{r}$ at 9.0 s:

(4) $$R=\frac{\varepsilon_{\max}-\varepsilon_{r}}{\varepsilon_{\max}}\times 100\%.$$

Meanwhile, the ratio of residual strain to peak strain was used to characterize the degree of unrecoverable deformation:

(5) $$P_{\mathrm{nr}}=\frac{\varepsilon_{r}}{\varepsilon_{\max}}\times 100\%.$$

**Figure 9:**
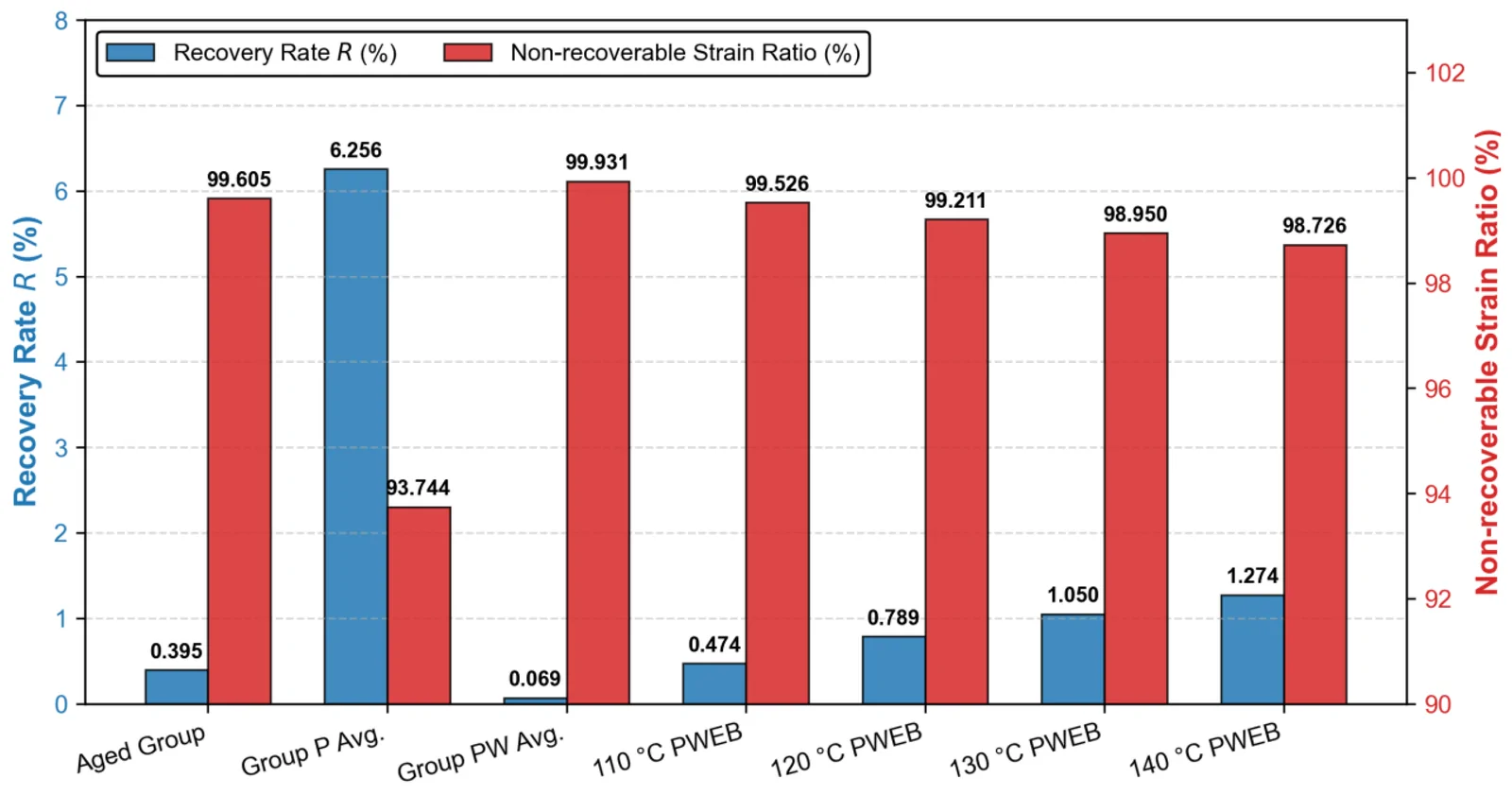
Comparison of characteristic parameters of high-temperature creep–recovery behavior of different asphalt binder groups at 64 °C.

As shown in Figure 9, which compares the characteristic parameters of high-temperature creep–recovery behavior of different asphalt binder groups at 64 °C, clear differences were observed among the groups. The peak strain and residual strain of the aged group were 2567.47% and 2557.33%, respectively, and the recovery rate was only 0.395%. These results characterize a highly rigid binder with very limited strain recovery, showing that aging-induced stiffness was accompanied by weak elastic recovery under repeated creep loading.

For the P group, the peak strain and residual strain decreased to 482.11% and 451.95%, respectively, and the recovery rate increased to 6.256%. The lower peak and residual strains together with the higher recovery rate indicate improved resistance to accumulated deformation and a greater recoverable component after PREW treatment.

In the PW group, the peak strain and residual strain increased to 9707.23% and 9700.54%, respectively, while the recovery rate was 0.069%. The high accumulated strain and low recovery rate characterize a strongly viscous deformation response after WCO incorporation. The enhanced molecular mobility that improves construction flowability therefore corresponds to greater susceptibility to permanent deformation at 64 °C.

Among the PWEB groups treated at different temperatures, the peak strain and residual strain of the 110 °C group reached 17,772.70% and 17,688.50%, respectively, with a recovery rate of 0.474%. These values indicate a highly deformable state with limited elastic recovery at the lower treatment temperature. The peak strain and residual strain of the 120 °C group were 9733.51% and 9656.74%, respectively, and the recovery rate was 0.789%. Relative to the 110 °C group, the lower accumulated strain and higher recovery rate indicate improved control of high-temperature creep deformation.

For the 130 °C group, the peak strain and residual strain further increased to 43,755.80% and 43,751.70%, respectively, and the recovery rate was 0.009%. The extremely high accumulated strain and very small recoverable fraction indicate the strongest viscous creep response among the investigated PWEB groups at 64 °C. This result also illustrates that the favorable BBR response observed at 130 °C corresponds to a different high-temperature deformation behavior, highlighting the trade-off between low- and high-temperature rheological properties.

The recovery rate of the 140 °C group increased to 1.274%, and the unrecoverable strain ratio decreased to 98.726%. The increased recovery fraction indicates greater recoverable deformation relative to the 130 °C group. Combined with its lower ductility and reduced low-temperature *m* value, the 140 °C treatment represents a shift toward a more constrained high-temperature response accompanied by reduced low-temperature relaxation.

Overall, the MSCR results reveal a strong dependence of creep–recovery behavior on the rejuvenation treatment condition. PREW treatment produced the lowest accumulated strain and the highest recovery rate among the P and PW stages, whereas WCO promoted a highly mobile and deformation-prone state. Among the PWEB temperature groups, the 120 °C treatment provided lower accumulated deformation than the 110 °C and 130 °C groups while retaining a measurable recovery response. Together with the BBR and DSR results, this behavior supports the selection of 120 °C as a comparatively balanced treatment condition rather than the optimum condition for every individual rheological indicator.

### 3.3. Microscopic Morphology and Chemical Structure Analysis

#### 3.3.1. FTIR Analysis

**Figure 10:**
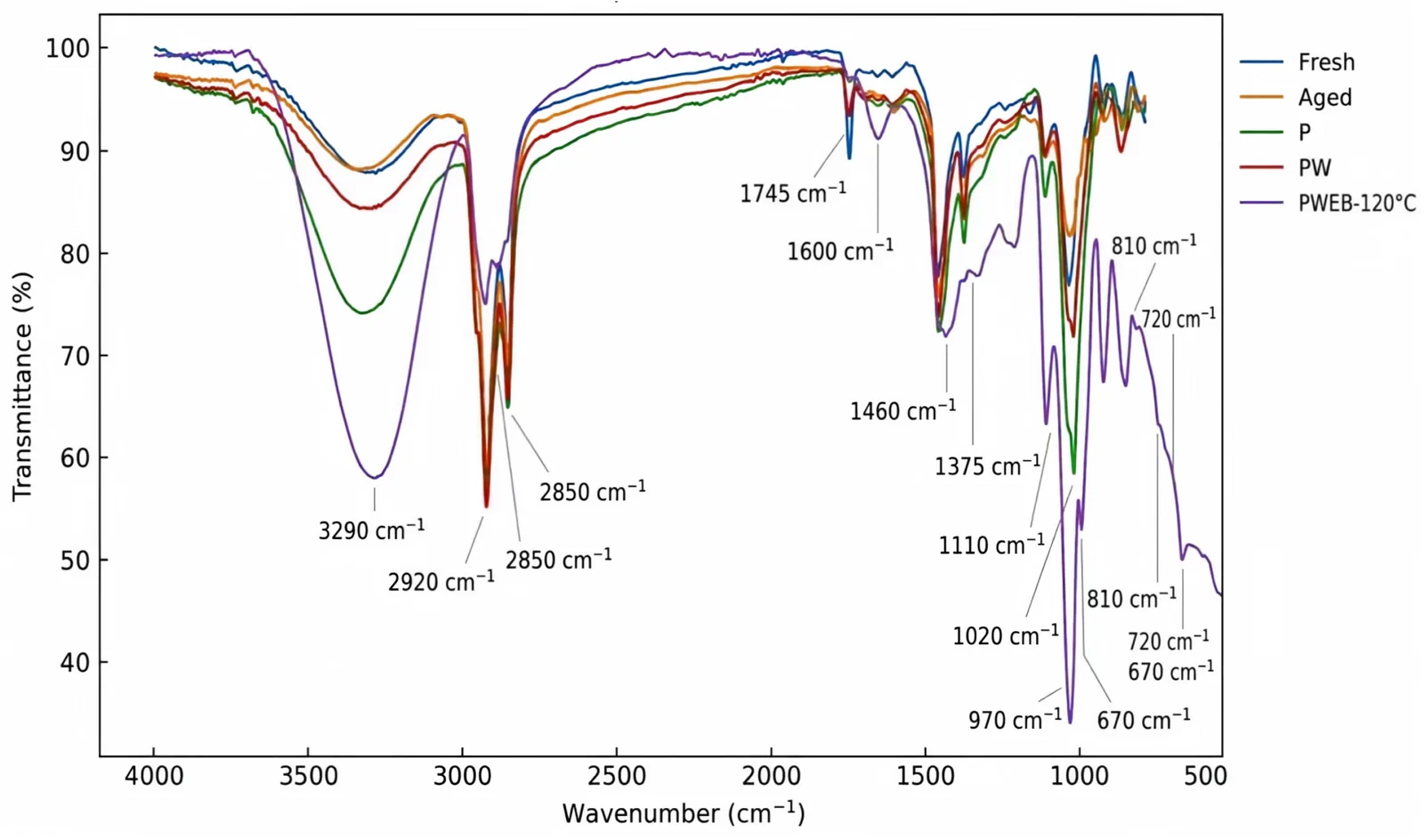
FTIR spectra of asphalt binders at different rejuvenation stages.

To further analyze the chemical structural changes in the composite rejuvenation system during the rejuvenation process, FTIR tests were conducted on asphalt binder samples from different groups. As shown in Figure 10, the FTIR spectra of asphalt binders at different rejuvenation stages exhibited typical characteristic absorption peaks of asphalt materials, including the broad peak near 3400 cm^−1^, the aliphatic C–H stretching vibration peaks near 2920 cm^−1^ and 2850 cm^−1^, the carbonyl-related absorption peak near 1740 cm^−1^, the aromatic ring skeleton vibration peak near 1600 cm^−1^, and the C–O-related absorption region within 1100–1000 cm^−1^. Compared with the fresh group, the aged group showed noticeable changes in the absorption characteristics of oxygen-containing functional group-related bands, indicating that thermal oxidative aging induced oxidation reactions and molecular structural evolution in the SBS-modified asphalt binder. The spectral changes of the P group relative to the aged group were relatively small, suggesting that PREW treatment was mainly associated with changes in the physical state of the aged matrix, accompanied by relatively limited variation in the FTIR absorption characteristics. After WCO was added, the intensity of some characteristic absorption regions in the PW group further changed, reflecting changes in the chemical environment associated with the incorporation of the oil-rich component and the increased mobility of the binder system. Overall, the FTIR response at this stage remained relatively moderate compared with that of the subsequent PWEB treatment.

Compared with the P and PW groups, the PWEB group exhibited more pronounced spectral changes in oxygen-containing functional group-related regions after the introduction of ESO and BDMA, especially in the broad peak region and the absorption range of 1100–1000 cm^−1^. These spectral variations indicate changes in the chemical environment of oxygen-containing functional groups during the ESO/BDMA treatment stage and are consistent with enhanced physicochemical interactions within the composite system.

**Figure 11:**
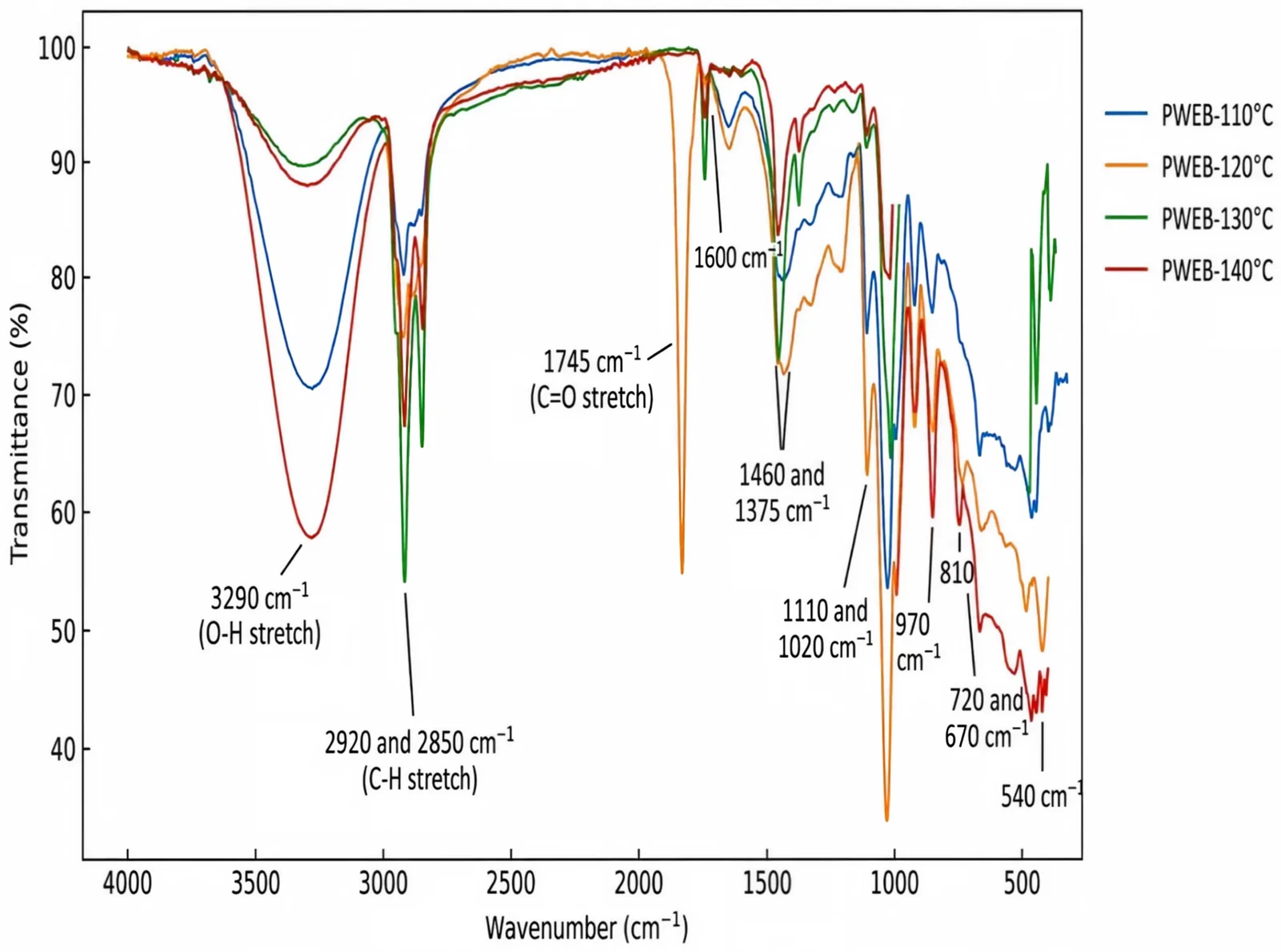
FTIR spectra of PWEB asphalt binders treated at different temperatures.

As shown in Figure 11, the FTIR spectra of the PWEB groups treated at different temperatures showed certain differences, indicating that temperature had a noticeable influence on the spectral response of the composite rejuvenation system. The spectral characteristics of the 110 °C group were generally close to those after physical softening, suggesting a comparatively moderate change in the oxygen-containing functional group-related regions at the lower treatment temperature. When the rejuvenation temperature increased to 120 °C, the changes in oxygen-containing functional group-related absorption regions became more evident, indicating a stronger temperature-dependent change in the local chemical environment of the composite system. When the temperature further increased to 130 °C and 140 °C, some characteristic regions still showed strong responses. The variation among these spectra followed a non-monotonic temperature-dependent pattern, suggesting that the chemical environment of the binder components evolved differently across the investigated treatment temperatures.

Overall, the FTIR results demonstrate qualitative changes in the absorption regions associated with oxygen-containing functional groups during the progressive rejuvenation process. PREW and WCO produced comparatively moderate spectral responses, whereas the introduction of ESO/BDMA was accompanied by more evident changes in these absorption regions. The temperature-dependent FTIR response of the PWEB groups was consistent with the evolution observed in the macroscopic and rheological properties.

#### 3.3.2. Fluorescence Microscopy Morphology Analysis

**Figure 12:**
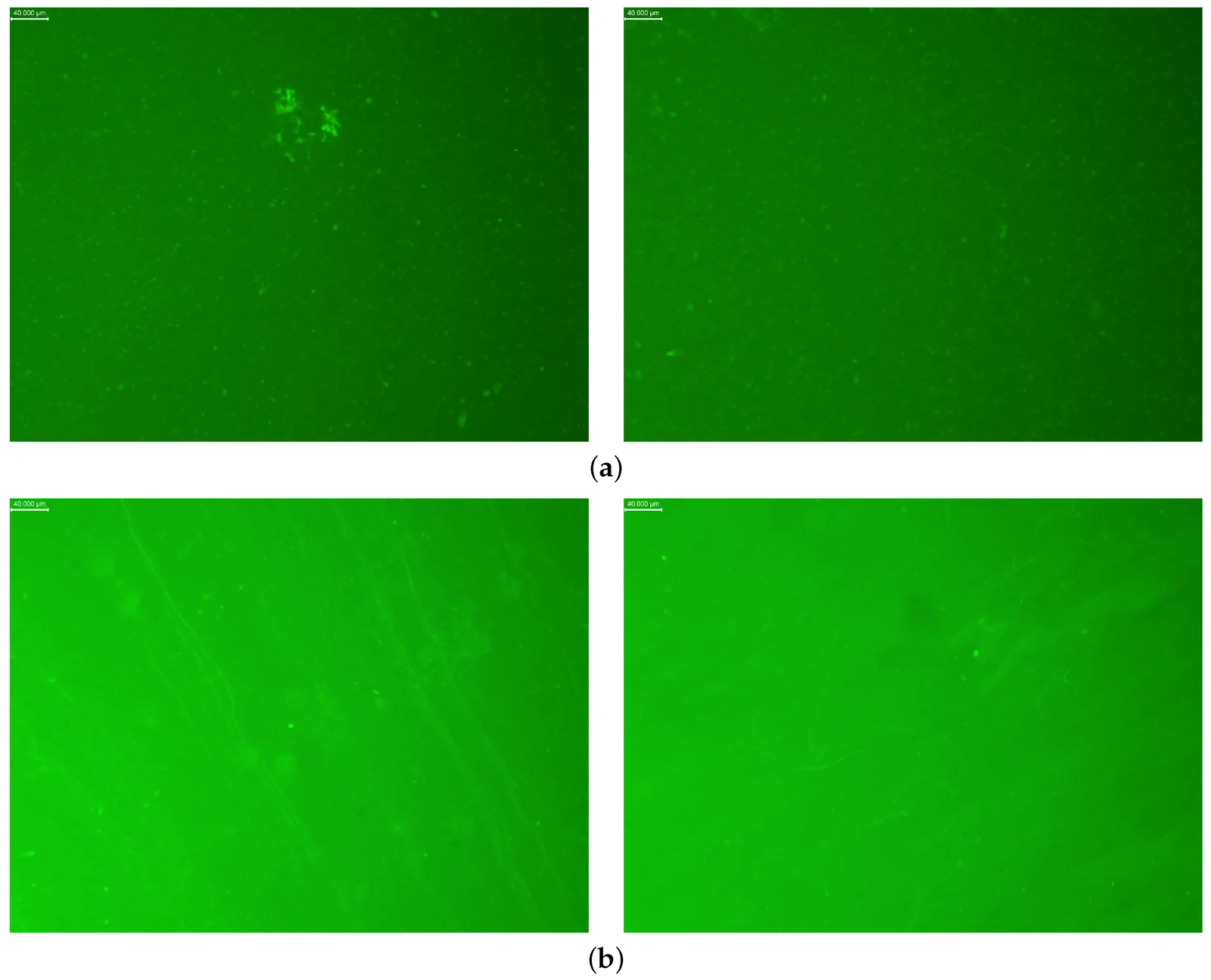

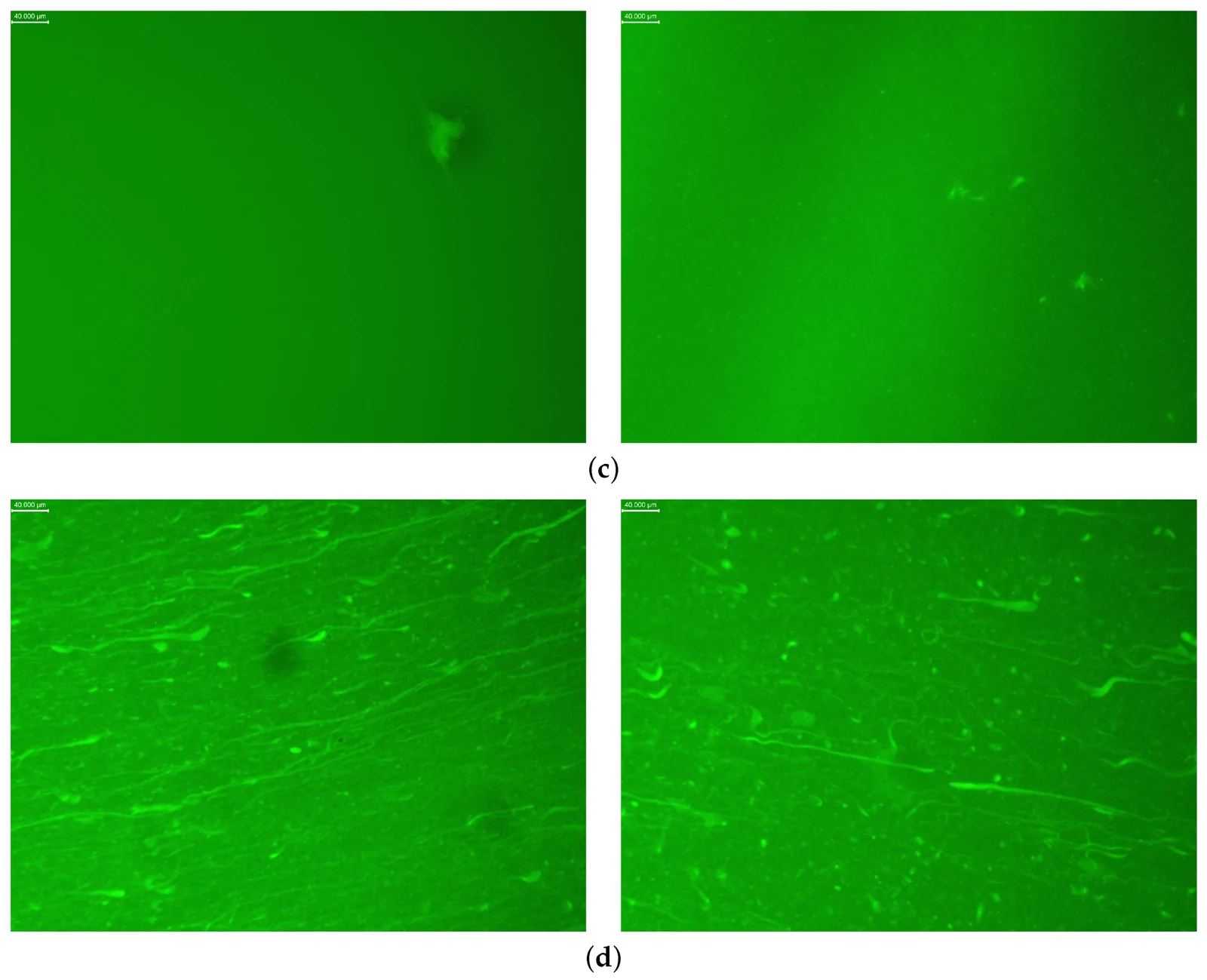
Fluorescence microscopy images of different asphalt binder groups: (**a**) aged group; (**b**) P group; (**c**) PW group; and (**d**) PWEB group. Each row contains two parallel fluorescence microscopy images of the same group.

The fluorescence microscopy results are shown in Figure 12. In the aged group, the fluorescent phase was mainly distributed as discrete dots and fine particles. The bright phases were isolated from each other, showing poor continuity, and obvious local aggregation could also be observed. These morphological features indicate that aging was associated with pronounced fragmentation, reduced apparent continuity, and local aggregation of the polymer-rich phase.

After PREW was added, the distribution of the fluorescent phase in the P group was slightly improved compared with that in the aged group. Some particles became larger, and the dispersion uniformity was enhanced to some extent. The overall morphology remained dominated by isolated particles and short fragments, while the apparent continuity of the polymer-rich phase showed only a moderate increase. This suggests that PREW treatment was associated mainly with adjustment of the matrix state and limited morphological evolution of the polymer-rich phase.

After WCO was added, the boundary of the fluorescent phase in the PW group became blurred, the particle size further increased, and a certain degree of swelling appeared in local regions. These features are consistent with enhanced mobility and redistribution of the polymer-rich phase within the softened asphalt matrix. The bright domains remained predominantly dispersed, with localized enlargement and increased interaction among adjacent domains. The resulting morphology reflects further physical-state adjustment of the polymer-rich phase after WCO incorporation.

The PWEB group showed more evident morphological evolution. The fluorescent phase gradually transformed from discrete particles into elongated, connected, and locally network-like structures, and the connectivity among the bright phases was clearly enhanced. These observations indicate increased apparent continuity and connectivity of the polymer-rich phase after ESO/BDMA treatment and are consistent with a more integrated phase morphology within the composite binder.

### 3.4. Multi-Indicator Comprehensive Balance Analysis

Based on the results of conventional properties, rotational viscosity, BBR low-temperature rheology, creep recovery at 64 °C, fluorescence microscopy, and FTIR, the PWEB composite rejuvenator system exhibited multifunctional regulatory behavior in aged SBS-modified asphalt binder. The progressive grouping results indicate that PREW mainly reduced the flow resistance of the aged system and was associated with the retention of relatively high-temperature structural resistance. WCO mainly enhanced binder mobility, flexibility, and low-temperature stress relaxation capacity, while the corresponding decrease in softening point reflected a shift toward a more softened high-temperature state. The introduction of ESO/BDMA enabled the PWEB group to show a more coordinated evolution among softening point, rotational viscosity, ductility, and high- and low-temperature rheological behavior. This pathway is consistent with recent findings on reactive rejuvenation, targeted composite rejuvenation, and dual-function rejuvenators.

**(1) Effects of PREW/WCO on the flowability and flexibility of the aged matrix**

The results of the P and PW groups show that PREW and WCO jointly established the physical regulation stage of the aged matrix. In the P group, the penetration recovered from 21.0 to 28.3, and the rotational viscosity at 135 °C decreased from 3560 mPa·s to 2280 mPa·s, indicating that PREW first reduced the flow resistance of the aged system. Meanwhile, the relatively high softening point was retained, reflecting the maintenance of a comparatively stiff high-temperature physical state after PREW treatment. After WCO was further introduced into the PW group, the penetration increased to 71.4, the creep stiffness (*S*) at −12 °C decreased to 184 MPa, and the *m*-value increased to 0.336, indicating enhanced flexibility and low-temperature stress relaxation capacity after WCO incorporation. Its softening point decreased to 63.3 °C, and the rotational viscosity at 135 °C was 224 mPa·s. These changes characterize a highly softened and mobile binder state, accompanied by reduced high-temperature structural resistance.

The FTIR results also support this observation. The spectra of the P and PW groups changed only slightly compared with that of the aged group, showing comparatively moderate variations in the characteristic absorption regions during the PREW/WCO treatment stage. Previous studies have reported that the light components in WCO can weaken interactions among asphaltenes through a solvation effect and increase the free volume of the system, thereby improving molecular mobility [22,35]. Combined with the results of this study, the PREW-related response is consistent with initial flow regulation, while WCO further increases molecular mobility and provides a more favorable environment for the dispersion of subsequent components.

**(2) Further effects of ESO/BDMA on the structural state of the composite rejuvenation system**

After ESO and BDMA were introduced on the basis of PREW and WCO pretreatment, the PWEB group exhibited performance and morphological characteristics distinct from those of the PW group. Taking the PWEB-120 °C group as an example, its penetration was 69.8, softening point was 72.1 °C, ductility reached 20.8 cm, and rotational viscosity at 135 °C recovered to 835 mPa·s. These results characterize a transition from the strongly softened PW state toward a more coordinated combination of flexibility, viscosity, and thermal resistance. The high-temperature creep recovery results also showed that the residual strain of the PWEB-120 °C group was slightly lower than that of the PW group, and its recovery rate was higher than those of the PWEB-110 °C and PWEB-130 °C groups. Under fluorescence microscopy, the polymer-rich phase in the PWEB group evolved from discrete particles to connected and elongated structures, with clearly enhanced apparent continuity and connectivity.

At the chemical structural level, the FTIR spectra of the PWEB group showed more pronounced changes in the broad band region and in the absorption range of 1100–1000 cm^−1^ associated with oxygen-containing functional groups, indicating changes in the local chemical environment during the ESO/BDMA treatment stage. Related studies have shown that epoxy-type rejuvenators can undergo ring-opening reactions with active groups in aged systems under tertiary amine catalysis, which is beneficial for reconnecting broken SBS chain segments [13,32]. Meanwhile, targeted composite rejuvenation studies have indicated that light-component replenishment can facilitate the diffusion and interaction of reactive components within aged asphalt systems [36]. Accordingly, the results of this study suggest that ESO/BDMA treatment is associated with further physicochemical adjustment of the composite system in the relatively mobile environment established by PREW and WCO. The FTIR and fluorescence observations support changes in chemical environment and polymer-rich-phase morphology, while the specific molecular reaction pathway requires complementary chemical characterization.

**(3) Influence of rejuvenation temperature on the performance balance of the composite rejuvenator**

Rejuvenation temperature influenced the relative contributions of component mobility, diffusion, and temperature-dependent physicochemical interactions in the composite system. Although the penetration of the PWEB-110 °C group reached 74.2, its softening point was only 68.4 °C, and the *m*-values at −12 °C and −18 °C were 0.298 and 0.276, respectively. These results characterize a relatively soft and mobile state together with limited low-temperature stress relaxation at this treatment temperature. The PWEB-120 °C group showed a comparatively coordinated combination of conventional properties, rotational viscosity, and high- and low-temperature rheological performance, suggesting that the physicochemical state established at this temperature provided a favorable balance among the evaluated properties.

The PWEB-130 °C group showed the best performance in terms of individual BBR parameters, but its peak strain and residual strain in creep recovery at 64 °C were substantially higher, showing a clear trade-off between favorable low-temperature relaxation and high-temperature creep resistance. For the PWEB-140 °C group, the softening point further increased to 76.8 °C, while the ductility decreased to 14.0 cm, and the *m*-value at −18 °C decreased to 0.297, indicating a shift toward a more constrained material state with reduced low-temperature flexibility.

The temperature-dependent evolution of FTIR spectra provided additional information on changes in the chemical environment of the composite system. The spectral characteristics at 110 °C showed comparatively moderate changes in the oxygen-containing functional group-related regions; at 120 °C, the absorption bands associated with oxygen-containing functional groups changed noticeably, showing a stronger temperature-dependent spectral response; whereas at 130 °C and 140 °C, some characteristic bands still showed strong changes, with a non-monotonic evolution across the investigated temperature range. Previous studies have indicated that reactive rejuvenator systems exhibit temperature-dependent interactions and that increasing thermal exposure can influence light-component mobility and the structural state of aged asphalt [10,16,17,37]. Combined with the results of this study, within the PWEB system and temperature range investigated here, the PWEB-120 °C group achieved a favorable comprehensive balance among conventional properties, rotational viscosity, high- and low-temperature rheological behavior, and polymer-rich-phase morphology.

## 4. Conclusions

Based on the analysis of conventional properties, rotational viscosity, low-temperature rheology, high-temperature creep recovery, fluorescence microscopy morphology, and FTIR spectra of aged SBS-modified asphalt binder and different rejuvenated asphalt binder groups, the following conclusions can be drawn.

1. After combined RTFOT and PAV aging, the penetration, ductility, and low-temperature *m*-value of the SBS-modified asphalt binder decreased markedly, while the softening point and rotational viscosity increased. This indicates that the binder exhibited pronounced hardening and embrittlement under the aging conditions used in this study, accompanied by weakened low-temperature stress relaxation capacity.
2. PREW pretreatment reduced the flow resistance of the aged asphalt binder to some extent and retained a relatively high softening point, indicating that it modified the physical state of the aged matrix. After WCO was added, the penetration and low-temperature rheological indices were improved, whereas the softening point and rotational viscosity decreased markedly. These results show that WCO addition enhanced binder flexibility and flowability while shifting the material toward a more softened high-temperature state.
3. After ESO and BDMA were introduced on the basis of PREW and WCO pretreatment, the PWEB group showed a more coordinated evolution among conventional properties, rotational viscosity, BBR parameters, and high-temperature creep recovery behavior. Within the temperature range investigated in this study, the PWEB-120 °C group exhibited the most favorable comprehensive balance among the evaluated properties. This treatment condition combined suitable construction flowability with comparatively coordinated low-temperature flexibility and high-temperature deformation resistance.
4. The fluorescence microscopy results showed that the polymer-rich phase in the PWEB group evolved from discrete particles to a more continuous morphology, with increased apparent continuity and connectivity of the fluorescent domains. This morphological evolution was consistent with the coordinated changes observed in the conventional and rheological properties of the PWEB system.
5. FTIR analysis revealed qualitative changes in the absorption regions associated with oxygen-containing functional groups during the progressive rejuvenation process. PREW and WCO treatment produced comparatively moderate spectral variations, whereas the introduction of ESO and BDMA was accompanied by more evident changes in these absorption regions. The PWEB groups also exhibited a temperature-dependent spectral response, with the 120 °C group showing FTIR characteristics consistent with its comparatively balanced macroscopic and rheological performance.

The present study focuses on binder-scale performance, rheological behavior, FTIR spectral characteristics, and fluorescence morphology. Future studies should integrate quantitative FTIR indices with complementary chemical characterization techniques, such as GPC, XPS, NMR, or Raman spectroscopy, to further clarify the molecular interactions associated with ESO/BDMA treatment and the respective contributions of PREW and WCO. Long-term aging, cyclic loading, and asphalt-mixture-scale tests will further support the evaluation of the durability and engineering applicability of the proposed composite rejuvenation system.

## Figures and Tables

**Table 1:** Basic properties of the SBS-modified asphalt binder.

Index	Test Value	Requirement
Penetration at 25 °C/0.1 mm	62.5	60–80
Softening point/°C	77.0	≥55
Ductility at 5 °C/cm	28.0	≥20
Rotational viscosity at 135 °C/mPa·s	1350	≤3000

**Table 2:** Aging test conditions.

Aging Method	Test Temperature/°C	Test Pressure/MPa	Test Duration
RTFOT	163	Atmospheric pressure	85 min
PAV	100	2.1	20 h

**Table 3:** Experimental groups and mix designs.

Group	PREW/%	WCO/%	ESO/%	BDMA/%	Rejuvenation Temperature/°C
Fresh group	0	0	0	0	0
Aged group	0	0	0	0	0
P group	3.0	0	0	0	115
PW group	3.0	8.0	0	0	115
110 °C PWEB group	3.0	8.0	5.0	0.5	110
120 °C PWEB group	3.0	8.0	5.0	0.5	120
130 °C PWEB group	3.0	8.0	5.0	0.5	130
140 °C PWEB group	3.0	8.0	5.0	0.5	140

**Table 4:** Conventional properties of different groups expressed as mean ± SD.

Group	Penetration at 25 °C/0.1 mm	Softening Point/°C	Ductility at 5 °C/cm
Fresh group	62.5 ± 1.8	77.0 ± 0.7	28.0 ± 1.1
Aged group	21.0 ± 1.1	86.0 ± 0.9	0.5 ± 0.2
P group	28.3 ± 1.3	81.4 ± 0.8	1.3 ± 0.3
PW group	71.4 ± 2.0	63.3 ± 0.6	10.7 ± 0.8
110 °C PWEB group	74.2 ± 2.2	68.4 ± 0.8	17.6 ± 0.9
120 °C PWEB group	69.8 ± 1.7	72.1 ± 0.7	20.8 ± 1.0
130 °C PWEB group	64.8 ± 1.5	74.8 ± 0.6	18.6 ± 0.8
140 °C PWEB group	59.5 ± 1.6	76.8 ± 0.8	14.0 ± 0.7

## Data Availability

The original contributions presented in the study are included in the article, further inquiries can be directed to the corresponding author.

## Abbreviations

The following abbreviations are used in this manuscript:

SBS	Styrene–butadiene–styrene
PREW	Recycled polyethylene wax
WCO	Waste cooking oil
ESO	Epoxidized soybean oil
BDMA	N,N-dimethylbenzylamine
PWEB	PREW/WCO/ESO-BDMA composite rejuvenator system
RTFOT	Rolling thin-film oven test
PAV	Pressure aging vessel
BBR	Bending beam rheometer
DSR	Dynamic shear rheometer
MSCR	Multiple stress creep recovery
FTIR	Fourier transform infrared spectroscopy
GPC	Gel permeation chromatography
